# Metatranscriptomic Investigation of Adaptation in NO and N_2_O Production From a Lab-Scale Nitrification Process Upon Repeated Exposure to Anoxic–Aerobic Cycling

**DOI:** 10.3389/fmicb.2018.03012

**Published:** 2018-12-06

**Authors:** Ariane Coelho Brotto, Medini K Annavajhala, Kartik Chandran

**Affiliations:** Department of Earth and Environmental Engineering, Columbia University, New York, NY, United States

**Keywords:** nitrous oxide, nitric oxide, biological nitrogen removal, metagenomics, metatranscriptomics, RNA-seq, adaptation

## Abstract

The molecular mechanisms of microbial adaptation to repeated anoxic–aerobic cycling were investigated by integrating whole community gene expression (metatranscriptomics) and physiological responses, including the production of nitric (NO) and nitrous (N_2_O) oxides. Anoxic–aerobic cycling was imposed for 17 days in a lab-scale full-nitrification mixed culture system. Prior to cycling, NO and N_2_O levels were sustained at 0.097 ± 0.006 and 0.054 ± 0.019 ppmv, respectively. Once the anoxic–aerobic cycling was initiated, peak emissions were highest on the first day (9.8 and 1.3 ppmv, respectively). By the end of day 17, NO production returned to pre-cycling levels (a peak of 0.12 ± 0.007 ppmv), while N_2_O production reached a new baseline (a peak of 0.32 ± 0.05 ppmv), one order of magnitude higher than steady-state conditions. Concurrently, post-cycling transcription of *nor*BQ and *nos*Z returned to pre-cycling levels after an initial 5.7- and 9.5-fold increase, while *nir*K remained significantly expressed (1.6-fold) for the duration of and after cycling conditions. The imbalance in *nir*K and *nos*Z mRNA abundance coupled with continuous conversion of NO to N_2_O might explain the elevated post-cycling baseline for N_2_O. Metatranscriptomic investigation notably indicated possible NO production by NOB under anoxic–aerobic cycling through a significant increase in *nir*K expression. Opposing effects on AOB (down-regulation) and NOB (up-regulation) CO_2_ fixation were observed, suggesting that nitrifying bacteria are differently impacted by anoxic–aerobic cycling. Genes encoding the terminal oxidase of the electron transport chain (*cco*NP, *cox*BC) were the most significantly transcribed, highlighting a hitherto unexplored pathway to manage high electron fluxes resulting from increased ammonia oxidation rates, and leading to overall, increased NO and N_2_O production. In sum, this study identified underlying metabolic processes and mechanisms contributing to NO and N_2_O production through a systems-level interrogation, which revealed the differential ability of specific microbial groups to adapt to sustained operational conditions in engineered biological nitrogen removal processes.

## Introduction

Sequential anoxic–aerobic cycling is commonly employed in biological nitrogen removal (BNR) wastewater treatment systems to promote integration of nitrification (oxidation of ammonia to nitrate) and denitrification (reduction of nitrate to nitrogen gas). However, this cycling process can lead to the production of nitric oxide (NO) and nitrous oxide (N_2_O), which are major ozone-depleting substances and the latter also a potent greenhouse gas (GHG) (Intergovernmental Panel on Climate Change [IPCC], 2007; Ravishankara et al., 2009). Indeed, there are now numerous studies reporting evidence of NO and N_2_O production by autotrophic ammonia-oxidizing bacteria (AOB) and denitrifying ordinary heterotrophic organisms (OHO) resulting from transient changes in dissolved oxygen (DO) concentration (Yu et al., 2010; Ahn et al., 2011; Chandran et al., 2011; Brotto et al., 2015; Soler-Jofra et al., 2018; Yu et al., 2018). These gasses are produced as a response to the resulting imbalance between supply and availability of electrons or reducing equivalents, as well as a shift in AOB and OHO metabolism toward a maximum specific substrate consumption (q_max_) or specific growth (μ_max_) rates.

Previous studies exploring the magnitude and mechanisms of nitrogenous-GHGs (N-GHG) emissions from BNR systems with respect to changes in DO concentration have imposed either a *single* transient condition (Yu et al., 2010; Brotto et al., 2015) or sustained limiting DO concentrations (Ahn et al., 2011), which might overestimate the magnitude of N-GHGs production. On the other hand, understanding the impact of *repeated* exposure to transient DO is particularly significant in the case of engineered systems, where microorganisms are continuously subjected to such sustained anoxic–aerobic cycling and yet these have been rarely examined. A steady decline in N_2_O emissions was previously observed upon repeated imposition of ammonia pulse-loadings to a pure *Nitrosomonas europaea* chemostat culture, pointing to potential adaptive responses in N_2_O production by AOB (Chandran et al., 2011). A recent study also reported adaptation of a pure culture of *N. europaea* ATCC 19718 to anoxic–aerobic cycling in terms of the proteome, whole-cell and reactor performance, and allowed for the modeling of NO and N_2_O production across multiple metabolic pathways (Yu et al., 2018). However, such adaptive responses have not been evaluated in mixed microbial communities, which could foster multiple organisms and pathways that contribute to NO and N_2_O production and could in turn display differential adaptation. Beyond a fundamental perspective, it is also important to consider and study the adaptation of nitrifying bacteria and OHO to anoxic–aerobic cycling, especially while investigating strategies to mitigate N_2_O production from wastewater treatment plants or while comparing the N_2_O footprints of different operational configurations.

Pathways involved in the production of N-intermediates from BNR processes have been investigated through chemical measurements (Kampschreur et al., 2008a,b, 2011; Ahn et al., 2010a,b; Foley et al., 2010; Lu and Chandran, 2010; Brotto et al., 2015), isotopic signature (Toyoda et al., 2011; Wunderlin et al., 2013), and proteomics (Lu et al., 2012; Jiang et al., 2015; Yu et al., 2018). Additionally, RT-qPCR (Yu et al., 2010; Ahn et al., 2011) has been used to target genes encoding key enzymes in nitrification and denitrification [ammonia monooxygenase (*amo*), hydroxylamine oxidoreductase (*hao*), nitrite reductase (*nir)*, nitrate reductase (*nar)*, nitric oxide reductase (*nor)*, and nitrous oxide reductase (*nos*)] (Graham et al., 2011). In a pure culture study of AOB (*N. europaea* ATCC 19718) before and after a *single* anoxic–aerobic cycle, an investigation of gene transcript concentrations of *amo*, *hao*, *nir*K, and *nor*B showed an increased expression of *nir*K, supporting the utilization of nitrite as an alternate electron acceptor under anoxic conditions, along with a reduction in the expression of *amo* and *hao*, likely to conserve cellular resources (Yu et al., 2010). However, while focusing on specific target genes offers useful information to characterize the pathways and mechanisms of N transformation, it cannot provide insight into a system-wide response, or to correlations and potential interplays across several metabolic pathways and different microbial communities than harbor them. For a more detailed interrogation, a systems biology approach is needed to look at changes in overall community structure (metagenomics) and expressed RNA-level activity (metatranscriptomics). These methodologies are superior to conventional targeted techniques since they allow for study at the biological systems level, rather than narrowly focusing on individual organisms or genes.

It is hypothesized here that sustained anoxic–aerobic cycling triggers adaptation in terms of microbial ecology and expression of N-cycle genes such as *nar, nir, nor* and *nos*, leading to an initial spike in NO and N_2_O emissions upon imposition of cycling followed by a gradual return to reduced or baseline levels. Additionally, the underlying electron imbalance causing these emissions suggests that adaptation can also be expected in terms of up- and down-regulation of genes involved in the flow of electrons in energy generation (e.g., oxidative phosphorylation). Finally, we hypothesize that bacteria can manipulate the relative expression of catabolic pathways (e.g., N metabolism) and anabolic pathways (e.g., fixation of inorganic carbon used for cellular growth) under alternating anoxic–aerobic conditions to better manage the associated electron flow, which in turn will minimize the production of NO and N_2_O. The application of metatranscriptomics to this study allows for the simultaneous exploration of these metabolic pathways and more, and can lead to a more thorough understanding of the molecular responses associated with microbial adaptation of a mixed culture nitrifying community to repeated imposition of anoxic–aerobic conditions.

## Materials and Methods

### Bioreactor Operation

**FIGURE 1:**
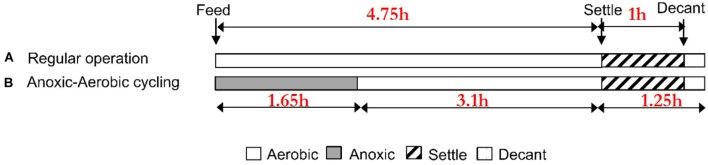
Description of SBR cycles for **(A)** regular operation and **(B)** anoxic–aerobic cycling.

A mixed nitrifying sequencing batch reactor (SBR, *V* = 12.5 L) was operated with an influent ammonia concentration of 40 mg N/L for 125 days in full-nitrification (oxidation of ammonia to nitrate) mode. Additionally, the SBR feed medium contained (per liter): 0.25 g of MgSO_4_ ● 7H_2_O, 0.10 g KH_2_PO_4_, 0.26 g K_2_HPO_4_ ● 3H_2_O, 0.35 g NaHCO_3_, 1 mL of trace elements solution [3.3 mg of FeSO_4_ ● H_2_O, 3.3 mg MnSO_4_ ● H_2_O, 0.67 mg (NH_4_)_4_Mo_7_O_24_ ● 4H_2_O, 0.83 mg CuCl_2_ ● 2H_2_O, 2.9 mg ZnSO_4_ ● 7H_2_O, 0.57 NiSO_4_ ● 6H_2_O in a total volume of 100 mL with distilled water]. The SBR had four 6-h cycles per day, each comprised of 3.5 h aerobic fill and react, 1.25 h aerobic react, 1 h settle, and 0.25 h decant phases (Figure 1A). SBR phases were automatically controlled via a digital controller (Chrontrol, Corp., San Diego, CA, United States). The bioreactor was operated at room temperature (21 ± 1.0°C), with a solids retention time (SRT) of 8 days, hydraulic retention time (HRT) of 1.25 days and target pH automatically controlled at 7.5 ± 0.10. Accordingly, the net nitrogen feeding rate during the aerobic fill and react cycles of the SBR cycle was 0.40 g N/day with a flowrate of 10 L/day. Aeration was provided by laboratory air filtered through a 0.2 um cartridge filter (Millipore^®^, Ann Arbor, MI, United States) at a flow rate of 2 L/min, and added mixing was provided by magnetic stirring.

### Experimental Design of Anoxic–Aerobic Cycling

Reactor biomass was subjected to transient anoxic periods (by switching from lab-air to N_2_ gas at the same total gas flow rate) of 1.65 h during the fill and react phase, followed by an aerobic period of 3.1 h in each of the four cycles per day for a duration of 17 days (Figure 1B). This experimental design of the anoxic–aerobic cycling of the SBR approximates operation of full-scale activated sludge processes, where usually 1/3 of the hydraulic residence time is anoxic and 2/3 is aerobic. Nitrogen transformations were monitored in terms of ammonia (NH_3_, colorimetry, Hach Method 10023, Hach Chemical, Co., Loveland, CO, United States), nitrite (${NO}_{2}^{-}$, diazotization – colorimetry) (Eaton et al., 2005), and nitrate (${NO}_{3}^{-}$, Dionex ICS 2100 Ion Chromatograph, Thermo Scientific, Inc., Waltham, MA, United States) every 50 min during anoxia (3 sampling points) and every 60 min during aerobic conditions (3 sampling points) for one 6-h cycle per day over 17 days. Once the imposition of anoxic–aerobic cycling concluded on day 17, we allowed another 20 days, which is 2.5 times the 8 days operating SRT, by which time, the transient responses had subsided even further, for collecting some terminal samples.

Reactor biomass concentrations were approximated using total chemical oxygen demand (tCOD, Hach Chemical, Co., Loveland, CO, United States) measurements. DO concentrations were measured in real-time using a polarographic Clark type electrode (YSI 5331A, Yellow Springs Instruments, Yellow Springs, OH, United States), connected to a dual-channel DO meter (YSI 5300) and interfaced to a personal computer. Online data acquisition was performed using virtual instrument codes implemented on LABVIEW, version 8.0 (National Instruments, Austin, TX, United States). Gaseous N_2_O and NO concentrations were detected in real-time using a USEPA-reviewed protocol (Chandran, 2011), employing infrared gas-filter correlation and chemiluminescence (Teledyne, San Diego, CA, United States), respectively, at a frequency of 1 per minute. The fraction of influent nitrogen emitted as N_2_O and NO was determined by numerically integrating the real-time profile of N_2_O and NO emission mass fluxes and normalizing to the influent ammonia N load, as described previously (Brotto et al., 2015).

### Biokinetics Estimation

Biokinetics of ammonia and nitrite oxidation were estimated via a previously described extant respirometric technique (Chandran and Smets, 2000). Respirometric assays were initiated by a sequential spike of nitrite (5 mg N/L) followed by ammonia (6 mg N/L) and were performed under air saturation (8–9 mg O_2_/L). Biokinetic parameters were expressed as the maximum specific oxygen uptake rate for ammonia oxidation (sOUR_nh_) and nitrite oxidation (sOUR_no2_) by normalizing the maximum slope of the volumetric oxygen uptake rate profiles to total biomass concentration (tCOD) as follows:

(1) $${sOUR}_{nh}=\frac{\left({dO}_{2}/{dt}_{\max,nh+no2}\right)}{X_{t}}-{sOUR}_{no2}$$

(2) $${sOUR}_{no2}=\frac{\left({dO}_{2}/{dt}_{\max,no2}\right)}{X_{t}}$$

Where dtO_2_/dt_max_ is the maximum oxygen uptake rate computed from the slope of a given respirograms (mg O_2_/L/h) and X_t_ is the total biomass concentration (g tCOD/L).

### Biomass Collection, DNA and RNA Extraction

Biomass samples from the SBR were collected every 50 min during anoxic conditions and 60 min during aerobic conditions (coincident with chemical sampling time points) for one of the four 6-h cycles each day and stored at -80°C for subsequent molecular processing and analysis. Total RNA samples were additionally treated with RNAprotect bacteria reagent prior to storage (Qiagen, Valencia, CA, United States). Total RNA samples from days 0 (steady-state), 1, 7, 17, and 38 (post-experiment) were extracted separately and extracts were subsequently combined for each day for further RNA-sequencing. Consequently, metatranscriptomics results display a composite response for each selected day and not for every time point sampled. DNA and RNA were extracted and prepared for sequencing as described in the Supplementary Material.

### Metagenomic and Metatranscriptomic Library Preparation and Analysis

Metagenomic and metatranscriptomics library preparation and sequencing on Ion Torrent PGM^TM^ (Life Technologies, Grand Island, NY, United States) was carried out as described in the Supplementary Material. For all metagenomic and metatranscriptomic libraries, raw reads were filtered for quality and minimum length using Mothur software ver. 1.34.4 (Schloss et al., 2009). Filtered reads were assembled using different assembler packages (SPAdes, SOAPdenovo2, and MIRA) (Chevreux et al., 1999; Luo et al., 2012; Nurk et al., 2013). Protein-coding features were predicted on the assembled metagenome contigs (Hyatt et al., 2010). A detailed description of bioinformatics, community identification, functional analysis and differential gene expression statistics can be found in the Supplementary Material. The workflow of the bioinformatics analyses is described in Supplementary Figure SI-1 and all raw sequence data from this study have deposited in the NCBI BioProject under accession number PRJNA291769.

## Results and Discussion

### Effects of Anoxic–Aerobic Cycling on N Intermediates and Biokinetics of AOB and NOB

**FIGURE 2:**
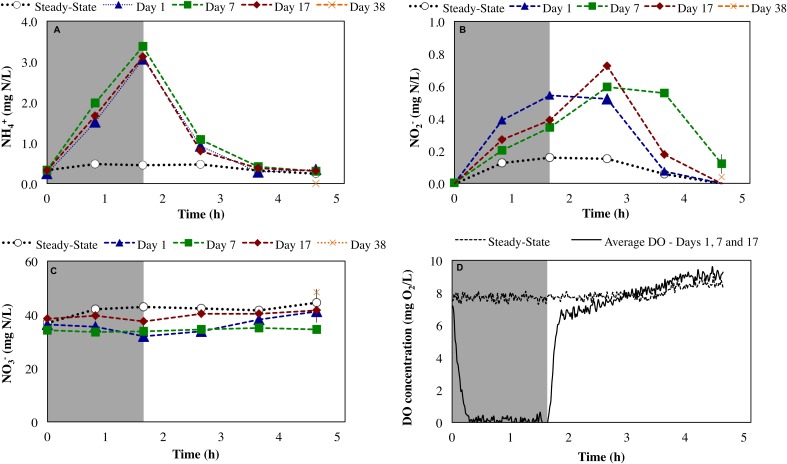
Impact of sustained transient anoxia on **(A)** ammonia, **(B)** nitrite, and **(C)** nitrate concentrations (all as mg N/L), and **(D)** DO concentrations (mg O_2_/L) relative to steady-state aerobic operation. Error bars represent the standard deviation of duplicate measurements. Shaded and unshaded areas represent anoxic and aerobic phases, respectively.

During steady-state operation of the mixed culture nitrifying SBR, preceding the repeated imposition of anoxic phases, the effluent ${NH}_{4}^{+}$, ${NO}_{2}^{-}$, and ${NO}_{3}^{-}$ concentrations were 0.13 ± 0.17, 0.12 ± 0.051, and 36 ± 6.9 mg N/L, respectively (*n* = 82). The extent of ammonia conversion was 99 ± 0.60%, primarily to nitrate (96 ± 0.24% infl-N) with low levels of residual nitrite (0.32 ± 0.13% infl-N). Gaseous NO and N_2_O concentrations at steady-state were 0.097 ± 0.006 ppmv (0.067 ± 0.004% infl-N) and 0.054 ± 0.019 ppmv (0.068 ± 0.024% infl-N), respectively. The reactor biomass concentration was 139 ± 94 mg COD/L. DO concentration was maintained in the range of 7.6 ± 0.66 mg O_2_/L (Figure 2D). The biokinetics of ammonia to nitrite oxidation (sOUR_nh_) and nitrite to nitrate oxidation (sOUR_no2_) were 30 ± 4 and 19 ± 1.8 mg O_2_/g tCOD/h, respectively.

Imposition of transiently anoxic conditions resulted in an expected, although not instantaneous decrease in DO concentrations (Figure 2D). During this period of decreasing DO concentrations at the initiation of the anoxic cycle, it is possible that both aerobic and anoxic transformations were occurring. Direct measurements of the reactor oxidation–reduction potential (ORP) could have been provided better insights into the extent of anoxia during the imposed anoxic–aerobic cycling. However, this was not the focus herein.

**FIGURE 3:**
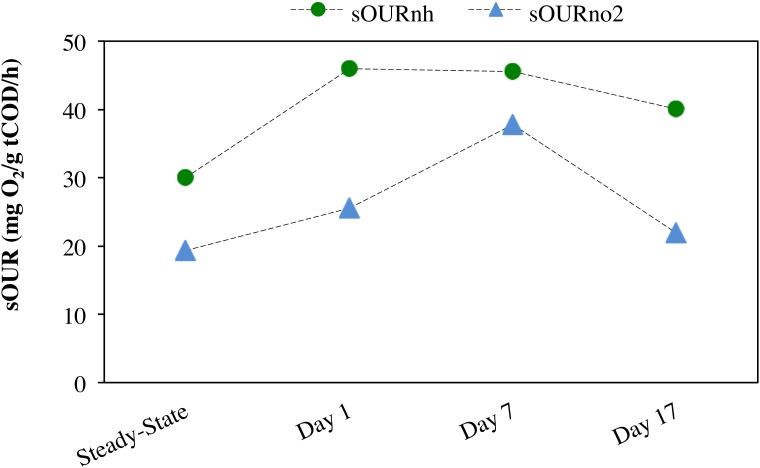
Specific oxygen uptake rate (sOUR) associated with ammonia (sOUR_nh_) and nitrite (sOUR_no2_) oxidation during steady-state aerobic and sustained anoxic–aerobic cycling.

Anoxic–aerobic cycling resulted in an accumulation of ammonia to an average of 2.9 ± 0.10 mg N/L in the anoxic phases of days 1, 7 and 17, due to reduction in ammonia oxidation activity by AOB (Figure 2A). However, no significant difference (*t*-test, α = 0.95) was observed in ammonia consumption rates of the anoxic phases (17 ± 0.44 mg N/g COD h) and post-anoxic phases (25 ± 8.8 mg N/g COD h) over the course of the study, resulting in similar ammonia profiles (shown only for days 1, 7 and 17, Figure 2A). Toward the end of each 6-h SBR cycle, the average ammonia concentrations during the cycling phase for day 1 to day 17 (0.36 ± 0.011 mg N/L) were statistically similar to the concentration levels during the pre-cycling steady-state conditions (0.32 ± 0.010 mg N/L) (Figure 2A). The biokinetics of ammonia oxidation (as inferred from sOUR_nh_ assays) increased from 30 mg O_2_/g tCOD/h during pre-cycling to 46 mg O_2_/g COD/h on day 1 and remained elevated throughout the 17 days of sustained anoxic–aerobic cycling (Figure 3). This indicates that the AOB respond to the shorter aerobic durations during the cycling phase by increasing their potential ammonia oxidation activity (sOUR_nh_) in order to consume oxygen and oxidize ammonia more efficiently.

Compared to the accumulation and subsequent removal of ammonia, removal of accumulated nitrite post-anoxia began more rapidly on day 1 (Figure 2B). Subsequently, within 2 h in non-limiting oxygen conditions, nitrite concentrations (0.074 ± 0.000 mg N/L) were statistically similar to pre-cycling steady-state levels (0.058 ± 0.001 mg N/L). For day 7 and day 17, on the other hand, nitrite concentrations were 10 and 3 times higher than pre-cycling concentrations, respectively, even 2 h after recovery to aerobic conditions (Figure 2B). In parallel, the biokinetics of NOB revealed a gradual increase of nitrite oxidation potential from day 1 to day 7, returning to pre-cycling levels by day 17 (Figure 3). In such a complex, mixed culture community, nitrite oxidation activity as inferred solely from NOB could be incomplete, for example due to dissimilatory nitrite reduction or denitrification by OHO (activity not measured for this system). However, these insights into the biokinetics of NOB do provide evidence for microorganism-level adaptation as a result of repeated exposure to anoxia. The markedly different responses of AOB and NOB to sustained anoxic–aerobic cycling highlights the heterogeneity of mixed culture nitrifying communities and the importance of understanding the effects of operational conditions on all protagonists in such a system, beyond those captured by just pure-culture studies (Yu and Chandran, 2010; Yu et al., 2010, 2018).

### Distinct Impacts of Sustained Transient Anoxia on NO and N_2_O Production

**FIGURE 4:**
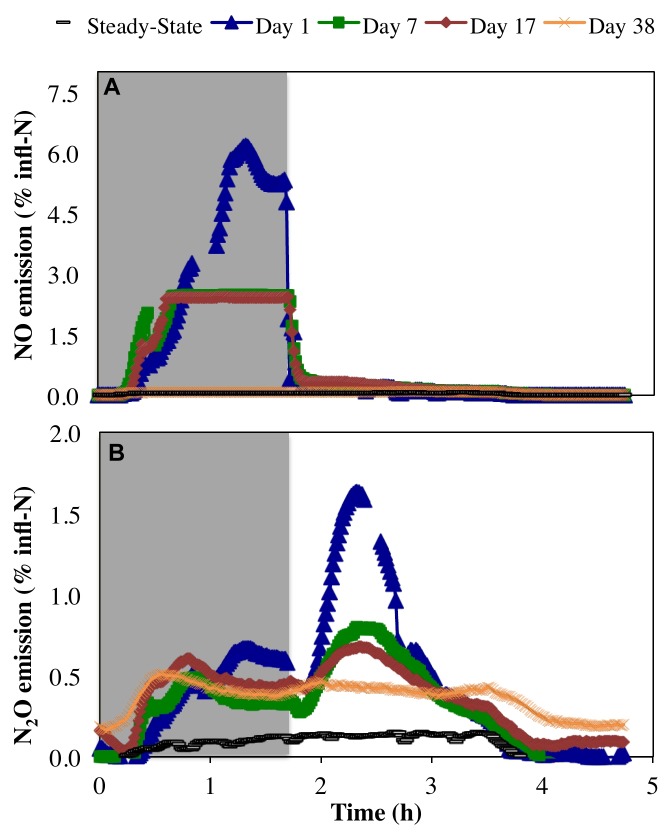
Relative proportion of N influent (% infl-N) converted to **(A)** NO and **(B)** N_2_O during steady-state aerobic and sustained anoxic–aerobic cycling.

Peak emissions of both NO and N_2_O were highest on day 1 of the anoxic–aerobic cycling (6.2 and 1.6% of total infl-N, respectively), followed by a substantial reduction by the end of the cycling conditions (2.4 and 0.68% infl-N, respectively) (Figure 4). During anoxia, both NO and N_2_O off-gas emissions were observed (Figure 4), with peak NO off-gas concentrations (9.7 ppmv) up to one order of magnitude higher than N_2_O off-gas concentrations emissions (0.53 ppmv during the anoxic period of day 1).

Previous pure culture results using *N. europaea* ATCC 19718 (a model ammonia oxidizing organism) revealed an initial spike in NO production upon imposition of anoxia during a *single* anoxic–aerobic cycle, and NO production was observed in the anoxic phase only (Yu et al., 2010). Here, NO production from a mixed culture was also primarily restricted to the anoxic phase (Figure 4). This is likely due to a combination of autotrophic and heterotrophic nitrogen reduction potentially supported by reducing equivalents from the cytochrome pool (Okabe et al., 2004). However, rather than immediately spiking upon imposition of anoxia, as seen in the pure culture of *N. europaea* (Yu et al., 2010), NO production increased before reaching a peak at the end of the anoxic phase (Figure 4A). This increase in NO production over the anoxic period might again be supported by continued autotrophic and heterotrophic activity during anoxia. The measured peak NO concentrations subsided over the course of anoxic cycling (day 1: 9.7 ppmv, day 7: 3.9 ppmv, day 17: 3.8 ppmv), leading ultimately to a complete reduction to pre-cycling values by day 38 (0.12 ± 0.007 ppmv). The autotrophic production of NO during the anoxic phase is supported by nitrifier denitrification and driven by a finite reserve intracellular reducing equivalents (Yu et al., 2010; Chandran et al., 2011; Klotz and Stein, 2011). The decline in AOB-driven NO production during anoxia can be attributed to the depletion of intracellular reducing equivalents, prior to adaptation to anoxic–aerobic cycling. Upon repeated anoxic–aerobic cycling AOB like become more adept at channeling the electrons through alternate pathways to minimize NO production (and both intra-and extra-cellular accumulation during anoxia) to minimize its toxic impacts (Yu et al., 2010).

Conversely, N_2_O generation under a *single* anoxic–aerobic cycle in the prior pure culture studies was restricted to aerobic conditions (Yu et al., 2010, 2018). However, anoxic N_2_O generation has been reported in a mixed nitrifying culture (Brotto et al., 2015) due to activity of OHO, albeit minimal compared to N_2_O produced upon recovery to aerobic conditions and consequent AOB activity. In this study, N_2_O production was observed during both anoxic and aerobic conditions, but was highest upon recovery to aerobic conditions due to oxidation of accumulated ammonia by AOB (Yu et al., 2010, 2018) (Figures 2A, 4B). During the course of cycling, peak N_2_O concentrations during the aerobic phase gradually decreased (day 1: 1.3 ppmv, day 7: 0.71 ppmv, day 17: 0.57 ppmv). Nevertheless, gaseous N_2_O concentrations did not return to steady-state levels even on day 38. In both anoxic and aerobic phases, off-gas N_2_O concentrations reached a plateau at 0.51 ± 0.05 ppmv, one order of magnitude higher than during pre-cycling conditions (0.054 ± 0.019 ppmv). The distinct profiles and longer-term changes in NO and N_2_O emissions suggest changes in the complex NO and N_2_O production pathways in multiple groups of organisms, as well as the differential capabilities of these organisms, including AOB, NOB, and OHO, to adapt over time. Additionally, these results stress that process changes in systems of any scale will have both short- and long-term effects, both of which must be studied to reveal the full extent of their impacts.

### Microbial Community Composition and Functional Classification

High-throughput shotgun metagenomic sequencing of the metagenome library generated a total of 676 Mbp of nucleotide sequences with 2,035,005 total filtered reads and 300 bp average read length after quality filtering (Table 1). Three software packages were used for *de novo* assembly including SPAdes, SOAPdenovo2 and MIRA. MIRA generated a total of 20,967 contigs, with higher total length of 26.2 Mbp and the highest N_50_ length compared to SPAdes and SOPdenovo2 (Supplementary Table SI-1). The average GC content was 56.5% and average depth-of-coverage of 23.3. In the MIRA-assembled metagenome contigs, 33,071 gene-coding regions were identified and translated to protein sequences using Prokka. Of the original 33,071 gene-coding regions, 4,633 were assigned a KO number by the KEGG Automated Annotation Server. In total, 2,202 unique KEGG functions were identified in the metagenome.

**Table 1:** Metagenomics and metatranscriptomic analysis statistics.

	Metagenome	Day 0	Day 1	Day 7	Day 17	Day 38
Total reads	2,253,400	1,610,657	3,209,929	2,326,633	1,901,403	2,874,643
Filtered reads	2,035,005	1,423,159	2,272,823	2,158,156	1,754,507	2,782,877
Assembled contigs	20,967					
Total contig length (bp)	26,247,372					
N50	1,802					
GC (%)	56.50					
Alignment onto contigs (%)	79.12					
Gene-coding regions	20X					
KEGG total	33,069					
KEGG unique	4,633					
Alignment (%)		70	71	77	75	76


**FIGURE 5:**
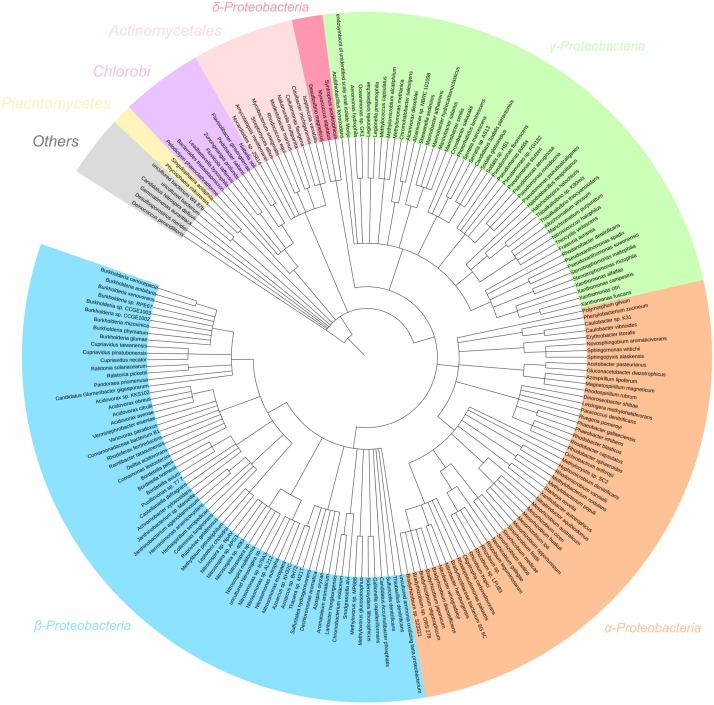
16S rRNA gene assignment of the filtered metagenome sequencing reads of the nitrifying reactor against NCBI 16S Microbial database using BLASTN search with E-value cutoff 1e-5.

In the metagenome, 97% of filtered reads were assigned to bacterial taxa (Figure 5). *Proteobacteria* was the most assigned phylum (92% of reads), comprised of reads assigned to α- (41%), β- (42%), γ- (8%), and *δ-Proteobacteria* (0.4%). Based on percent of total reads assigned, several key genera involved in nitrogen transformation were identified and considered for further interpretation: AOB (19% *Nitrosospira*, 7% *Nitrosomonas*, 0.1% *Nitrosococcus*), NOB (26% *Nitrobacter*, 0.1% *Nitrospira*), *nos*Z-capable heterotrophs (1% *Bradyrhizobium*, 1% *Rhodobacter*, 0.7% *Pseudomonas*, 0.6% *Rhizobium*) and dissimilatory nitrate reduction to ammonium (DNRA)-capable heterotrophs (0.1% *Thiobacillus*, 0.1% *Desulfovibrio*). The dominance of AOB genus *Nitrosospira* is due to their high affinity for substrate, thus commonly found in low-ammonia environments similar to the SBR reactor in operation (Schramm et al., 1999; Chain et al., 2003). Whereas, due to their higher maximum growth rates, *Nitrobacter*-related NOB have been shown to out-compete *Nitrospira* under non-limiting extant nitrite and DO concentrations experienced during anoxic–aerobic cycling conditions. The inoculum for the reactor in this study originated from a nitrifying reactor subject to a higher influent ammonia concentration (400 mg N/L) and operated at 8 days SRT for over 2 years (data not shown). The continuous operation of the SBR at a lower influent ammonia concentration (40 mg N/L) at the same SRT for this study was likely not enough to shift the microbial community to higher abundance of *Nitrospira*-related NOB. Other taxa not directly implicated on the production and/or consumption of the N species (especially NO and N_2_O) were not included as references for metatranscriptomic analyses for this study.

### Impacts of Sustained Anoxic–Aerobic Cycling on the Community Metatranscriptome

The underlying *in situ* microbial metabolism linking accumulation and subsequent oxidation of both ammonia and nitrite with NO and N_2_O production under transient anoxic–aerobic conditions were explored through metatranscriptomics at the community mRNA level. Here, gene expression profiles corresponding with nitrogen and carbon metabolism, as well as electron transport chain (ETC) pathways were investigated along with nitrogen redox transformations.

#### Impact on Nitrogen Metabolism

**FIGURE 6:**
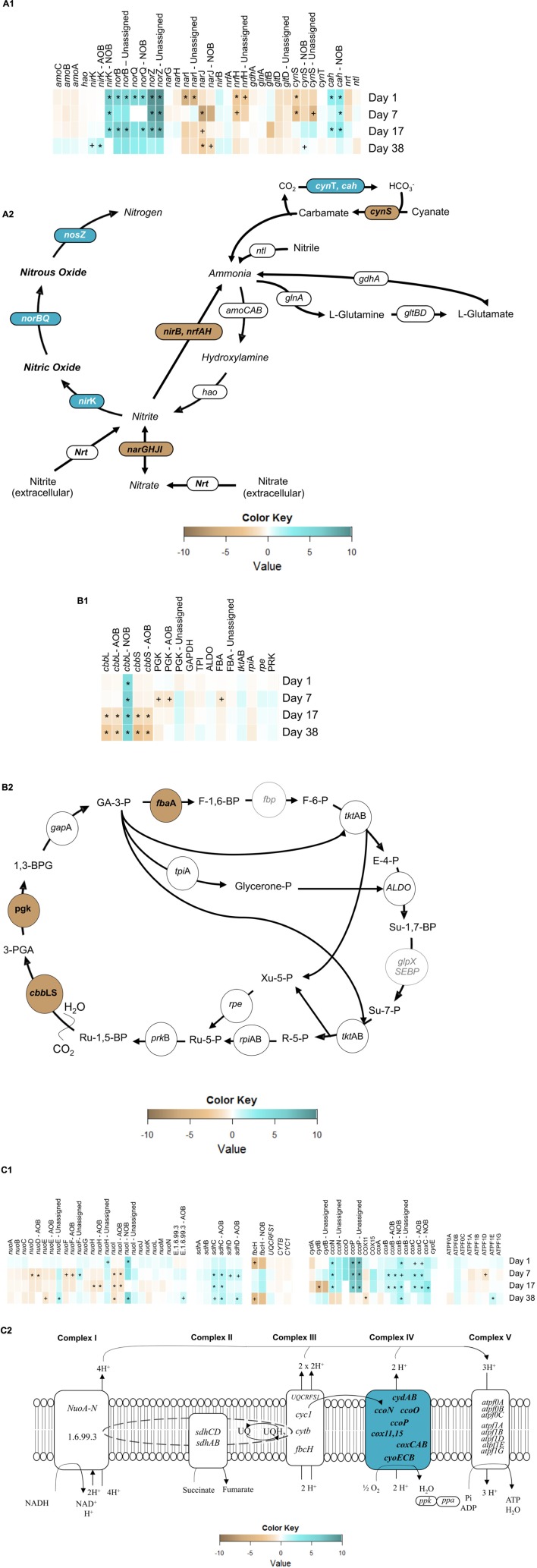
Heatmaps of gene expression pertaining to **(A1)** nitrogen metabolism, **(B1)** Calvin-Bassham-Benson cycle and **(C1)** electron transport chain (ETC) and, expression of overall reconstructed KEGG metabolic pathways of **(A2)** nitrogen metabolism, **(B2)** Calvin-Bassham-Benson cycle and **(C2)** electron transport chain. Symbols reflect statistically significant changes in gene expression: *^∗^p* < 0.05, *^+^p* < 0.10 in response to anoxic-aerobic cycling.

In autotrophic AOB catabolism, the oxidation of ammonia to hydroxylamine is catalyzed by ammonia monooxygenase (AMO), encoded by the *amoCAB* operon. AMO utilizes reducing equivalents produced from the oxidation of hydroxylamine to nitrite by hydroxylamine oxidoreductase (HAO), encoded by *hao* (Figure 6A2) (Zumft, 1997). There was no significant change (α = 0.95) in *amo*CAB or *hao* transcript levels from day 1 to day 38 (Figure 6A1). These results are in contrast to those of Yu et al. (2010), who previously observed an imbalance in *amo*A transcripts under a *single* transient anoxic–aerobic cycle in pure *N. europaea* cultures, with decrease in the expression during the anoxic phase and recovered upon recovery to aerobic conditions. However, lack of longer term impacts in average *amo* and *hao* expression per cycle in this present study points to the ability of the mixed AOB population herein to overcome sustained transients in nitrogen and electron fluxes due to differential ammonia oxidation rates and nitrite accumulation levels during and post-anoxia. Such an adaptive response is especially vital for the survival and performance of AOB in engineered systems, where the microbial community is constantly facing variations in the supply of electron donor and acceptor.

The two main classes of dissimilatory nitrite reductase, which produce NO from nitrite, are the heme-cytochrome *cd*1 type, coded for by *nirS*, and the copper-containing type, encoded by *nirK*, both of which exist among OHO (Hochstein and Tomlinson, 1988; Glockner et al., 1993; Cantera and Stein, 2007). Autotrophic AOB and NOB, on the other hand, only possess *nir*K (Hochstein and Tomlinson, 1988; Garbeva and Baggs, 2007). Three contigs in the community metagenome were annotated with *nir*K gene (Supplementary Table SI-3), one of which was aligned to AOB (86% similarity with *N. multiformis*) and another to NOB (97% similarity with *N. winogradskyi*), while the third contig remained unassigned yet appeared to be related to OHO. Transcript levels of AOB-related *nir*K, remained with no significant expression throughout the cycling conditions, but increased after the cessation of the anoxic–aerobic cycling (day 38, *p* = 1.9 × 10^-3^). The single cycle pure culture AOB study by Yu et al. (2010) reported an increase in *nir*K expression upon imposition of anoxia, followed by a decrease to steady-state levels upon recovery to aerobic conditions. Upon repeated anoxic–aerobic cycling, Yu et al. (2018) also observed increased *nirK* expression by *N. europaea* during anoxia, followed by reduction to steady-state levels, although expression of *nirK* upon recovery of aeration did decrease over the 13 day experiment. Since the mRNA library herein consisted of pooled RNA samples from each anoxic–aerobic cycle, it is possible that the overall expression of *nirK* was offset by opposing trends during anoxic and aerobic conditions, resulting in an unchanging average daily expression profile. Furthermore, *Nitrosospira multiformi*s rather than *Nitrosomonas europaea* represented the majority of AOB in this lab-scale reactor, likely due to the higher affinity for ammonia in the former (Schramm et al., 1999). Although *N. multiformis* has been shown to be capable of NO and N_2_O production through nitrifier denitrification (Shaw et al., 2006; Starkenburg et al., 2006), the impacts of transient anoxia or sustained operational conditions have not been previously evaluated for this organism. After cessation of the anoxic–aerobic cycling, the up-regulation of the *nirK* gene in AOB could explain the new baseline of N_2_O production—elevated compared to pre-cycling levels, by continuous conversion of NO. Interestingly, the metatranscriptomic investigation revealed an NOB imprint to the transcripts associated with NO production under anoxic–aerobic cycling. For NOB-related *nirK*, mRNA levels increased significantly during the anoxic–aerobic cycling for day 1 (*p* = 8.5 × 10^-5^), day 7 (*p* = 0.01), and day 17 (*p* = 0.04). Three genomes of NOB (*N. winogradskyi* Nb-255, *N. hamburgensis* X14, and *Nitrobacter* sp. Nb-311A) display a gene cluster encoding a putative *nir*K-type nitrite reductase (Freitag et al., 1987). Although NO has been reported as a terminal product of denitrification in anaerobic growth of *N. winogradskyi* (Starkenburg et al., 2008a), very little is known about the mechanisms of NO production by NOB. It has been previously reported that the putative *nir*K (nwi2648) in *N. winogradskyi* was transcribed under both aerobic (10–20% O_2_) and oxygen-limiting conditions (0–4% O_2_) in the presence of nitrite when growing lithoautotrophically (30 mM NaNO_2_) (Beaumont et al., 2004). Thus, the accumulation of nitrite during both anoxic and aerobic conditions herein (Figure 2B) likely contributed to the significant increase in expression of *nir*K and production of NO from NOB, although the latter cannot be confirmed. After cessation of transient oxygen conditions and a return to low nitrite concentrations, *nir*K transcripts from NOB also returned to pre-cycling levels (Figure 6A1). NO concentrations in the SBR reactor returned to pre-cycling levels by day 38 while overall *nirK* transcription was elevated post-cycling, suggesting that post-cycling recovery was reached faster at the metabolic level than at the transcription level.

NO is reduced to N_2_O by the cNOR complex, encoded by the *nor* gene cluster (Hendriks et al., 2000) with high homology between sequences in AOB and OHO (Hendriks et al., 2000). *norC* encodes a membrane-anchored cytochrome c-type that forms a complex with the major membrane-bound catalytic subunit, which is encoded by *norB* (Spiro, 2012; Simon and Klotz, 2013). *nor*Q is linked to *nor*CB as an accessory gene essential for the activation of NorCB (Hendriks et al., 2000; Miyahara et al., 2010). Contigs were annotated with *nor*B and *nor*Q genes. Transcripts of these genes displayed increased levels on day 1 (*p* = 0.011) of anoxic–aerobic cycling (Figure 6A1), likely in response to the observed increase in NO production (Hendriks et al., 2000) (Figure 4A), which in turn resulted in increased N_2_O production (Figure 4B). *nor*B and *nor*Q transcripts returned to pre-cycling levels in parallel with decreased levels of N_2_O production post-cycling compared to day 1, although emissions remained one order of magnitude higher than pre-cycling conditions (Figure 4B). For *nor*B, no taxonomic alignment could be identified, while the *no*rQ gene was aligned to a hypothetical protein in NOB (96% similarity with *N. winogradskyi*) (Supplementary Table SI-3). However, it has been previously reported that *N. winogradskyi* genome lacks predicted homologs of a NOR (Freitag et al., 1987), although N_2_O has been reported as a terminal product from respiratory nitrate reduction by *Nitrobacter* spp. with glycerol or pyruvate as electron donor (Starkenburg et al., 2008a). Thus, the direct role of NOB in the production of N_2_O is inconclusive, and such understanding would perhaps require further studies on pure cultures of NOB under similar growth and operational conditions.

Finally, the conversion of N_2_O to N_2_ is catalyzed by the copper-containing enzyme nitrous oxide reductase (Brandes et al., 2007), encoded by the *nos*Z gene. This is the only metabolic pathway for the consumption of N_2_O found in all extant canonical denitrifiers that produce N_2,_ and is also found in a few non-denitrifying bacteria that can use N_2_O as a terminal electron acceptor (Yoshinari, 1980; Hochstein and Tomlinson, 1988; Starkenburg et al., 2008b). Therefore, although no singular taxonomic alignment could be identified within the 80% cutoff identity (Supplementary Table SI-3) for the one contig identified as *nos*Z, *nos*Z transcription reported here can be inferred as related to OHO conversion of N_2_O to N_2_. Expression of *nos*Z was up-regulated (*p* < 0.05) throughout the sustained anoxic–aerobic cycling (Figure 6A1), gradually reducing from a 9.5-fold (day 1) to 8.7-fold (day 7) to 7.4-fold (day 17) increase compared to steady-state levels. The persistent up-regulation of *nos*Z is likely a response from the heterotrophic denitrifiers to compensate for the increased production of N_2_O during both anoxic and aerobic conditions, and convert the increasingly available N_2_O into N_2_. The return of *nos*Z mRNA levels to pre-cycling conditions on day 38 implies that OHO adapted to the prolonged anoxic–aerobic cycling through increased processing of higher N_2_O levels, but only up to a certain threshold, resulting in a plateau of N_2_O emissions post-cycling (Figure 4B). The findings in expression levels of *nir*, *nor*, and *nos* genes are especially interesting in their paralleling of observed N_2_O emissions—not trivial due to the potential for biological regulation at multiple points in transcription, translation, and post-translation—potentially making a case for these genes as biomarkers for N_2_O production. While the expression patterns reveal insights into the possible pathways or even protagonists involved in the production of N_2_O (or other metabolic intermediates and products), the physiological basis for the chemoorganoheterotrophic reduction of nitrogen species must also be considered. Although the reactor feed was entirely devoid of organic carbon, it is likely that at the operating SRT of 8 days, endogenous decay and release of intracellular organic carbon supported chemoorganotrophic growth and activity.

The *narGHJI* operon encodes for dissimilatory nitrate reduction, the first step of denitrification and is homologous to *nxr*AB (Freitag et al., 1987) in *Nitrobacter* spp. (Freitag et al., 1987; Kirstein and Bock, 1993; Arp et al., 2002). Unlike *nar*GH, *nar*J, and *nar*I were down-regulated (*p* < 0.05) during the transient oxygen cycling (Figure 6A1). For *nar*J the contig was aligned to NOB (95% similarity with *N. winogradskyi*) while for *nar*I no alignment could be identified (Supplementary Table SI-3). NarI is a b-type cytochrome that serves as the electron acceptor from the quinone pool and electron donor to the molybdenum cofactor in NarJ (Freitag et al., 1987). Transcript levels of *nar*J decreased for day 7 (*p* = 9.2 × 10^-5^) and 17 (*p* = 0.06) and remained at low levels by day 38 (*p* = 8.3 × 10^-3^) compared to pre-cycling conditions, while *nar*I levels decreased only on day 1 (*p* = 0.010), returning to steady-state levels afterward. The overall down-regulation of *nar*JI transcripts can be associated with either nitrite oxidation or nitrate reduction, yet the changing electron fluxes in the system are likely resulting in a shift of electrons away from their role in the reversible interconversion of nitrite and nitrate.

Discussions on expression profile of pathways involved in nitrogen assimilation and the DNRA are unlike to contribute to NO and N_2_O production during anoxic–aerobic cycling. Nevertheless, these are presented in the Supplementary Material for the sake of completeness.

#### Impact on Inorganic Carbon Fixation Through the Calvin-Benson-Bassham (CBB) Cycle

The CBB cycle mediates carbon fixation to support autotrophic growth of both AOB and NOB (Bock, 1976; Wei et al., 2004). However, the effects of oxygen levels on *cbb* have only been studied for pure culture of *N. europaea* (English et al., 1992). CO_2_ fixation is catalyzed by the ribulose 1,5-bisphophate carboxylase-oxygenase enzyme (RuBisCO, coded by *cbbLS*) in various organisms, including the major AOB and NOB included in the metagenome herein (Figure 6B2). Additionally, several denitrifiers have been identified as having copies of *cbb*L and *cbb*S: *Thiobacillus denitrificans*, *Rhodobacter capsulatus*, and *Rhodobacter sphaeroides* (Paoli et al., 1998; Pereira et al., 2001).

One contig for *cbb*L and two contigs for *cbb*S were aligned with AOB (*N. multiformis* and *Nitrosomonas* sp. Is79A3) (Supplementary Table SI-3). Transcript levels of both *cbb*L and *cbb*S in AOB significantly decreased on day 17 (*p* = 0.02 and 2.2 × 10^-3^, respectively) and remained at significant lower levels even 3 weeks after the cessation of the anoxic–aerobic cycling (day 38, *p* = 8.6 × 10^-4^ and 3.9 × 10^-6^) (Figure 6B1). The decrease in abundance of *cbb*LS mRNA is in agreement with previous reports that showed lower transcription of *cbb*LS in response to constant oxygen-limiting conditions (English et al., 1992). For this study, the effect of oxygen limitation imposed by the transient anoxia on inorganic carbon fixation was only evident by the end of the anoxic–aerobic cycling, pointing to a long-term impact on the autotrophic AOB CO_2_ fixation. Accumulation of ammonia during transient anoxic conditions could lead to a lack of available reducing equivalents for carbon fixation, causing the AOB to de-prioritize expression of genes related to biosynthesis, including *cbbLS* (English et al., 1992).

One contig each for *cbb*L and *cbb*S were also aligned with NOB (97 and 98% similarity with *N. winogradskyi*, respectively). In contrast with the AOB response, transcript levels of NOB-related *cbb*L were significantly high (*p* < 0.05) throughout and after the anoxic–aerobic cycling (Supplementary Table SI-2) in comparison to pre-cycling transcript levels (Figure 6B1). Although AOB and some NOB (e.g., *Nitrobacter* spp.) both fix CO_2_ via CBB cycle for growth, these findings indicate that RuBisCO gene expression is regulated differently in these organisms under sustained anoxic–aerobic cycling. Linking back to the N-cycle intermediates profiles (Figure 2), AOB and NOB are clearly affected differently by the sustained changes in electron availability and nitrogen flux during cycling. This finding is supported here at the RNA level, as AOB appear to channel energy away from CO_2_ fixation, or cell growth, while the opposite trend is observed in NOB.

#### Impacts of Transient Electron Flow on the Electron Transport Chain (ETC)

Transcript expression patterns revealed distinct trends across the four complexes that comprise the ETC (Figure 6C2). The most highly expressed genes of the ETC code for *aa*_3_- (*p* < 10^-5^) and *cbb*_3_-type (*p* < 10^-16^) cytochrome c oxidases, which belong to the heme-copper oxidase (HCO) superfamily (Larsson et al., 1995) (ferrocytochrome-*c*:oxygen oxidoreductase, Complex IV, EC 1.9.3.1), the terminal enzyme of the aerobic and anaerobic respiration in AOB, NOB, and heterotrophs (van Verseveld et al., 1977). Cytochrome-c oxidase transduces electrons from cytochrome c or quinones to reduce oxygen (O_2_) to water (H_2_O) and uses the released energy to pump protons across the membrane, driving the synthesis of adenosine triphosphate (Wikstrom, 1977; Kumar and Nicholas, 1982; Pitcher and Watmough, 2004). Some genes encoding the *cbb*_3_-type cytochrome c oxidase, *cco*N and *cco*P, were up-regulated (*p* < 0.05) during the anoxic–aerobic cycling (Supplementary Table SI-2), while *cco*O remained at levels similar to pre-cycling conditions (Figure 6C1). Although it was not possible to infer which organisms were transcribing *cco*NOP, cytochrome *cbb*_3_ oxidases have been purified from several species of *Proteobacteria*, including those identified in the metagenome (Pitcher et al., 2002). The intense response of *cbb*_3_-type cytochrome c oxidase expression (*p* < 10^-16^) in response to continued anoxic–aerobic cycling might be explained by its fivefold higher oxygen affinity compared to that of *aa*_3_-type cytochrome oxidase (*cox*BC) (Perez-Garcia et al., 2014). *cbb*_3_-type cytochrome c oxidase has been reported as expressed predominantly under low oxygen concentrations (Perez-Garcia et al., 2014). Here, the limiting oxygen conditions imposed by sustained anoxic–aerobic cycling followed by high electron flow channeled through the ETC likely contributed to the up-regulation of *cbb*_3_-type cytochrome c oxidase. Using a stoichiometric metabolic network (SDN) model (Machado et al., 2006; Perez-Garcia et al., 2014) suggested that NO and N_2_O production during anoxic–aerobic transition by *N. europaea* is a result of electron overproduction by the *bc*1 complex relative to the capacity of the terminal oxidase (in their study, *aa*_3_-type) to use the produced electrons. Interestingly, here, genes encoding for the *bc*1 complex (ubiquinol:cytochrome c oxidoreductase, Complex III, EC 1.10.2.2) were not significantly expressed during or post-cycling. The sampling schedule/sample pooling strategy utilized may not have adequately captured the dynamic function of the *bc*1 complex at the mRNA level (i.e., oxidation of quinol and reduction of cytochrome c coupled with proton transfer). In addition, the possible rapid channeling of electrons to periplasmic cytochromes (e.g., cytochrome c552) and the terminal cytochrome c oxidase due to electron flow imbalances could explain the lack of cytochrome *bc*1 mRNA response and the increased NO and N_2_O production by *nir* and *nor*.

Previously, the expression and regulation of the selected genes on the ETC has been evaluated for pure cultures of anaerobe and facultative anaerobe microorganisms in the presence of oxygen (Cotter et al., 1990; Mouncey and Kaplan, 1998; Lobo et al., 2007). Here, the impact of anoxic–aerobic cycling on the ETC as a whole, in addition to carbon and nitrogen metabolism, and the implication on NO and N_2_O production has only been possible due to the integration of metagenomics and metatranscriptomics.

In sum, the use of metatranscriptomics, integrated with reactor-level measurements allowed for a systems view of a mixed culture nitrifying bioreactor subjected to repeated cycles of anoxic–aerobic transients. Despite initial peaks in NO and N_2_O production resulting from ammonia and nitrite accumulation, the reactor community displayed the capability to adapt to the reduced availability of oxygen as an electron acceptor and modulated mRNA expression in an attempt to survive under these changing conditions. Specifically, genes involved in the generation, transport, and utilization of electrons key to energy synthesis were affected in order to prioritize survival over biosynthesis. The impacts of sustained anoxic–aerobic cycling on multiple metabolic pathways are thus crucial to better understand not only short- but also long-term ramifications of similar process changes in engineered nitrogen cycle processes.

## Author Contributions

ACB, MKA, and KC contributed to conception and design of the study. ACB and MKA performed the experiments and data analyses. ACB interpreted the data and wrote the manuscript with contributions from MKA. All authors contributed to manuscript revision, and have read and approved the submitted version.

## Conflict of Interest Statement

The authors declare that the research was conducted in the absence of any commercial or financial relationships that could be construed as a potential conflict of interest.
