# Supplementary material for: Metatranscriptomic Investigation of Adaptation in NO and N2O Production From a Lab-Scale Nitrification Process Upon Repeated Exposure to Anoxic–Aerobic Cycling
Source: Front Microbiol. 2018 Dec 6;9:3012. doi: 10.3389/fmicb.2018.03012 (PMC6291752; doi:10.3389/fmicb.2018.03012)
Supplement: Supplementary file 1 [file Table_1.docx]

**Supplementary Material**

**Metatranscriptomic investigation of adaptation in NO and N_2_O production from a lab-scale nitrification process upon repeated exposure to anoxic-aerobic cycling**

Ariane Coelho Brotto^1§^, Medini K Annavajhala^1§^, Kartik Chandran^1*^

**^1^** Department of Earth and Environmental Engineering, Columbia University, New York, NY 10027, USA

*Correspondence:

Kartik Chandran

[kc2288@columbia.edu](mailto:kc2288@columbia.edu)

^§^ Contributed equally to this work

*Running title*: Metatranscriptomics of community adaptation to anoxic-aerobic cycling

**1. Materials and Methods**

**1.1 DNA and RNA extraction**

DNA extraction for biomass samples from Day 1, 7 and 17 was conducted using DNeasy mini kits, automated on a QIAcube instrument (Qiagen, CA). Extracted DNA was purified using QIAquick DNA Cleanup kits (Qiagen, CA) and the quality and quantity of DNA were assessed using a NanoDrop Lite Spectrophotometer (Thermofisher, MA). Based on preliminary molecular fingerprinting via PCR-denaturant gradient gel electrophoresis (PCR-DGGE), no changes in the community ecology were found throughout the experiment and therefore, purified DNA extracts were pooled with equal number of cells from each of the 3 days for whole-genome sequencing. Total RNA samples from Days 0 (steady-state), 1, 7, 17 and 38 (post-experiment) for 3 sampling points (2 during anoxia and 1 during aerobic conditions) were extracted separately using RNeasy mini kits (Qiagen, CA). Total RNA quality and quantity were assessed using a NanoDrop Lite Spectrophotometer (Thermofisher, MA). Total RNA extracts were subsequently combined for each day for further RNA-sequencing.

**1.2. Metagenomic and Metatranscriptomic libraries preparation and sequencing**

The genomic DNA library was prepared using the NEBnext Fast DNA Fragmentation and Library Prep Set for Ion Torrent (New England Biolabs, MA). Fragments of 400 bp size were separated and extracted with an E-Gel® SizeSelect™ 2% gel (Life Technologies, NY). To avoid any polyclonality of Ion Sphere Particles^TM^ (ISPs) during template preparation, library quantification was performed with the KAPA Library Quantification kit for Ion Torrent (KAPA Biosystems, MA). Template preparation using the DNA library, followed by ISP enrichment, was performed using the Ion OneTouch2 system as per the manufacturer’s instructions (Ion OT2 400 kit, Product No. 4479878). The enriched ISPs were loaded into an Ion Torrent 316v2 chip and sequenced according to the manufacturer’s instructions (Ion PGM™ Sequencing 400 Kit, Product No. 4482002).

External RNA Controls Consortium (ERCC) spike-in control mixes were added to the total RNA of the combined sample from each selected date according to the manufacturer’s instructions (Ambion, Life Technologies, Product No. 4455352). Subsequently, rRNA was removed using the Ribo-Zero Magnetic Kit for gram-negative bacteria (Epicentre, IL). Metatranscriptome libraries for each of the selected cycles (Day 0, Day 1, Day 7, Day 17, and Day 38) were prepared using the Ion Total RNA-Seq Kit v2 (Life Technologies, NY). Ion Xpress RNA-Seq barcodes (Life Technologies, NY) were added to each sample. Quantification and quality of each final cDNA library was obtained by smear analysis using the Agilent High Sensitivity DNA kit for Agilent 2100 Bioanalyzer (Agilent Technologies, CA). Template preparation with the cDNA libraries followed by ISP enrichment was performed using the Ion OneTouch2 system as per manufacturer’s instructions (Ion OT2 400 kit, Product No. 4479878). The enriched ISPs were loaded onto an Ion Torrent 318 chip and sequenced according to manufacturer’s instructions (Ion PGM™ Sequencing 400 Kit, Product No. 4482002).

**1.3. Community Identification and Functional Analysis of Metagenome**

Filtered metagenome reads were aligned to the NCBI nucleotide (nt) database using BLAST with a cutoff E-value of 1e-^20^ to characterize the microbial community. The list of species of interest identified with BLAST was used to create a reference organism list for the Kyoto Encyclopaedia of Genes and Genomes (1) assignment. Online KEGG Automated Annotation Service (2) was used to assign KEGG orthology (KO number) functions to each of these features predicted by Prodigal using the bi-directional best hit (BBH) GHOSTX protocol. Additionally, a taxonomic tree was constructed with MEGAN ver. 5.10.3 (3) and exported to the web-based tool Interactive Tree of Life (iTOL) (4) for metagenome visualization.

**1.4. Differential Gene Expression Analysis**

Filtered metatranscriptome reads were aligned to the protein-coding features of the metagenome using Bowtie2 package ver. 2.2.2.4 (5). Read counts for all the five metatranscriptome samples were calculated for each KEGG-assigned feature, filtered at a cutoff of 1 count per million (cpm) for at least two libraries and normalized to total library size. The edgeR package in R (ver. 3.1.2) (6) was used to perform differential gene expression (dge) analysis to determine which genes had been differentially expressed in Days 1, 7, 17, and 38 when compared to the baseline (Day 0) (Table SI-2). The gplots package in R (7) was used to create heatmaps showing log-fold change of each gene’s normalized read count for the selected days of the anoxic-aerobic cycling. The underlying mechanisms linking nitrogen transformation and the metatranscriptomics at the community mRNA level linked was evaluated in terms of carbon fixation (ko00710, ko00720), nitrogen metabolism (ko00910) and electron transport chain through the oxidative phosphorylation (ko00190). Metatranscriptome-level response of other carbon pathways, such as Glycolysis/Gluconeogenesis (k00010), tricarboxylic acid cycle (k00020) and pentose phosphate (k00030) are depicted in Figure SI-2.

Organisms contributing to the differential expression of key genes in the selected pathways were identified through further analysis. In each of the studied KEGG pathways, genes that were differentially expressed (p<0.05 and p<0.1) in at least one time-point (Day 1, Day 7, Day 17, or Day 38) when compared to baseline expression levels were identified. The metagenome features for these genes were then selected and aligned using BLAST (ver 2.2.31) to a custom database of NCBI Reference Sequences (RefSeq) including the genomes of AOB, NOB, nosZ- and DNRA-capable organisms found in the metagenome (Table SI-3). Cutoff identity of 80% and e-value of 1e^-20^ were used to assign each feature to a known reference organism with certainty. Read count data was then reassigned to each of these assigned features; the total read counts for each gene were split into groupings of reads assigned to AOB, NOB, Denitrifiers, and unassigned reads. Differential gene expression analysis was performed on each of these groupings (AOB, NOB, Denitrifiers, Unassigned) for the RefSeq-assigned genes to determine the contributions of each organism to the overall changes in expression at all time points.

**2. Results and Discussions**

**2.1. Nitrogen catabolism and anabolism**

During the dissimilatory nitrate/nitrite reduction to ammonia (DNRA), nitrite is converted to ammonia by periplasmic pentahaem nitrite reductase (*Nrf*) and cytoplasmic sirohaem enzyme (*NirB*). NrfH forms a stable complex with NrfA that has been solved from several *proteobacteria* including *Thiobacillus denitrificans* and *Desulfovibrio magneticus,* which were detected in the metagenome (8-10). Unlike NirB, which is cytoplasmic and utilizes NADH as reductant, NrfA and NrfH are periplasminc and encode for cytochrome c-552 and cytochrome c nitrite reductase. Transcripts of *nrf*A were not differentially expressed throughout the anoxic-aerobic cycling conditions, even though it has previously been identified as a key component in NO detoxification (11). On the contrary, *nrf*H showed decrease mRNA levels for Day 1 (p=0.048) and Day 7 (p=0.053), returning to steady-state levels by the end of the anoxic-aerobic experiment. One contig was mapped with *nrf*H gene but no significant match could be assigned. Even though NrfAH functions independently of oxygen (12), *it* has been shown decreased transcription abundance (4-FC) for *nrf*H in response to nitrite stress (2.5 mM sodium nitrite) (13). Nitrite concentration in this study was at lower level (0.61 ± 0.084 mg N/L), but still *nrf*H expression decrease was 2.6-fold and 2-fold for Day 1 and Day 7, respectively, likely in response to nitrite.

**2.2. Nitrogen assimilation**

In overall, fewer genes on the N anabolic pathways were differentially expressed compared to the genes on the N catabolic pathways in response to sustained anoxic-aerobic cycling. Possibly, as the majority of microorganisms in this system are nitrifying bacteria (ca. 60%) and rely on the N catabolic metabolism to obtain their energy for growth and maintenance via autotrophic chemosynthesis (14-17). It has been previously reported that *cyn*S is repressed by ammonium in two strains of cyanobacteria and that the levels of *cyn*S transcription is low in ammonium grown cells (16). During sustained anoxic-aerobic cycling it is likely that the accumulation of ammonium during anoxia spurred the decrease of *cyn*S transcripts, which extended even after cessation of the anoxic-aerobic cycling. Of the differentially expressed (p<0.05) are the genes encoding cyanate lyase (E.C. 4.2.1.104) and carbonic anhydrase (E.C 4.2.1.1).

The *cyn*S (cyanase) is part of an operon encoding for cyanase lyase which catalyses bicarbonate-dependent decomposition of cyanate (NCO^-^) to CO_2_ and ammonia (16-18). It has been shown to be present in some heterotrophic bacteria, including *Pseudomonas aeruginosa*, with expression induced by its substrate (19). Two contigs of *cyn*S gene were mapped to the metagenome, with only one alignment to NOB (96 % *N. winogradskyi*) (Table SI-3). In overall, lower mRNA levels of *cyn*S was observed for Day 1 (p=0.02) and Day 7 (p=2.6 x 10^-3^).

Carbonic anhydrase (CA), encoded by *cyn*T gene (first gene of the *cyn* operon) and *cah*, is a zinc-containing metalloenzyme that catalyses the interconversion of carbon dioxide (CO_2_) and bicarbonate (HCO_3_^-^) in many organisms. Only one contig was mapped with *cah* gene, aligning to with NOB (88% similarity with *N. hamburgensis*) (Table SI-3). Increased transcript levels of NOB-related *cah* was observed throughout the anoxic aerobic (3.4-FC on Day 1 to 2.1-FC on Day 7 and 3.4-FC on Day 17). Upon cessation of the experimentanoxic-aerobic cycling *cah* transcripts returned to pre-cycling levels. Previous studies on *N. europaea* have shown increased transcription of *cah* as a mechanism to assimilate low concentrations of CO_2_ (20, 21). Such an investigation approach has not been conducted for NOB so far, but these findings suggest increase in intracellular CO_2_ during anoxic-aerobic cycling. Further studies are needed to investigate the mechanism of *cah* expression and carbonic anhydrase activity on NOB under anoxic-aerobic cycling.

**Table SI-1**. Comparison of assembler generated from Quast (22).

|  | SPAdes | SOAPdenovo | MIRA |
| --- | --- | --- | --- |
| # contigs (>= 0 bp) | 12,203 | 12,724,85 | 20,967 |
| # contigs (>= 100 bp) | 6,181 | 3,247 | 5,923 |
| Largest contig | 358,075 | 7,387 | 1,42,151 |
| Total length (>= 0 bp) | 19,364,825 | 127,504,370 | 262,53,524 |
| Total length (>= 100 bp) | 14,307,057 | 4,328,927 | 16,767,787 |
| N50 | 1,610 | 713 | 1,802 |
| N75 | 984 | 594 | 853 |
| L50 | 2,412 | 9,891 | 2,469 |
| L75 | 6,395 | 17,445 | 7,789 |
| GC (%) | 59.51 | 56.45 | 56.5 |
| Coverage depth | 31.5 | 4.8 | 23.3 |

**Table SI-2.** p-values of the differential gene expression analysis for each experimental day of selected KEGG pathways.

| **KO** | **Gene** | **Product** | **Day 1** | **Day 7** | **Day 17** | **Day 38** | | |
| --- | --- | --- | --- | --- | --- | --- | --- | --- |
| **Nitrogen Metabolism** | | | | | | |  |  |
| K10946 | pmoC-amoC | methane/ammonia monooxygenase subunit C | 0.32860134 | 0.496922148 | 0.622954102 | 0.243271589 | | |
| K10944 | pmoA-amoA | methane/ammonia monooxygenase subunit A | 0.298664793 | 0.15821852 | 0.369444591 | 0.255892415 | | |
| K10945 | pmoB-amoB | methane/ammonia monooxygenase subunit B | 0.230689553 | 0.313015485 | 0.339968844 | 0.348280206 | | |
| K10535 | hao | hydroxylamine dehydrogenase | 0.859289779 | 0.785623415 | 0.682827853 | 0.761416795 | | |
| K00368 | nirK | nitrite reductase (NO-forming) | 0.87015871 | 0.902612848 | 0.732573818 | 0.088601471 | | |
| K04561 | norB | nitric oxide reductase subunit B | 0.011070506 | 0.304197761 | 0.002681523 | 0.180915616 | | |
| K04748 | norB | nitric oxide reductase subunit B | 0.029036932 | 1 | 0.131803056 | 0.33972429 | | |
| K00376 | nosZ | nitrous-oxide reductase | 2.56334E-24 | 9.35799E-21 | 3.33811E-15 | 0.152656734 | | |
| K00370 | narG | nitrate reductase alpha subunit | 0.830112116 | 0.395562414 | 0.690843966 | 0.876149259 | | |
| K00371 | narH | nitrate reductase beta subunit | 0.288239498 | 0.582304507 | 0.420483051 | 0.987562171 | | |
| K00374 | narI | nitrate reductase gamma subunit | 0.01058724 | 0.881312639 | 0.131480565 | 0.180114557 | | |
| K00373 | narJ | nitrate reductase delta subunit | 0.16703966 | 9.32938E-05 | 0.063669471 | 0.008318858 | | |
| K00362 | nirB | nitrite reductase (NADH) large subunit | 0.531693288 | 0.77705331 | 0.883894486 | 0.623548364 | | |
| K03385 | nrfA | nitrite reductase (cytochrome c-552) | 0.844710184 | 0.603117581 | 0.486244898 | 0.237234449 | | |
| K15876 | nrfH | cytochrome c nitrite reductase small subunit | 0.048174384 | 0.052960691 | 0.565407463 | 0.324051373 | | |
| K00262 | E1.4.1.4, gdhA | glutamate dehydrogenase (NADP+) | 0.358572875 | 0.941758171 | 0.775203482 | 0.895889031 | | |
| K01915 | glnA | glutamine synthetase | 0.707788923 | 0.766988325 | 0.888723413 | 0.441467086 | | |
| K00265 | gltB | glutamate synthase (NADPH/NADH) large chain | 0.800254406 | 0.77705331 | 0.209854714 | 0.719939892 | | |
| K00266 | gltD | glutamate synthase (NADPH/NADH) small chain | 0.387412741 | 0.404547289 | 0.464248268 | 0.622272419 | | |
| K01725 | cynS | cyanate lyase | 0.025199637 | 0.002626716 | 0.723691167 | 0.54046905 | | |
| K01673 | cynT | carbonic anhydrase | 0.987771133 | 0.666881075 | 0.785559397 | 0.593550325 | | |
| K01674 | cah | carbonic anhydrase | 2.2828E-06 | 0.251820399 | 0.00070795 | 0.720409085 | | |
| K02575 | NRT | MFS transporter, NNP family, nitrate/nitrite transporter | 0.137605671 | 0.290522832 | 0.459211021 | 0.797614901 | | |
| K01501 | E3.5.5.1, ntl | nitrilase | 0.548112577 | 0.565555699 | 0.972027114 | 0.146576223 | | |
| **Calvin-Benson-Bassham Cycle (CBB)** | | | | | | |  |  |
| K01601 | cbbL | ribulose-bisphosphate carboxylase large chain | 0.577262824 | 0.752886 | 0.018484333 | 3.71152E-06 | | |
| K01602 | cbbS | ribulose-bisphosphate carboxylase small chain | 0.46892487 | 0.36788154 | 0.001971479 | 6.14828E-09 | | |
| K00927 | PGK | phosphoglycerate kinase | 0.866728723 | 0.074938302 | 0.535857894 | 0.264735341 | | |
| K00134 | GAPDH | glyceraldehyde 3-phosphate dehydrogenase | 0.806695131 | 0.263306312 | 0.431495225 | 0.562658954 | | |
| K01803 | TPI | triosephosphate isomerase (TIM) | 0.565863077 | 0.481389079 | 0.876011556 | 0.399579239 | | |
| K01623 | ALDO | fructose-bisphosphate aldolase, class I | 0.929544491 | 0.475210628 | 0.745875289 | 0.792346839 | | |
| K01624 | FBA | fructose-bisphosphate aldolase, class II | 0.886483257 | 0.096113297 | 0.362075073 | 0.421482444 | | |
| K00615 | E2.2.1.1, tktAB | transketolase | 0.665734991 | 0.152506651 | 0.468529747 | 0.310901161 | | |
| K01807 | rpiA | ribose 5-phosphate isomerase A | 0.702621308 | 0.623144131 | 0.292351797 | 0.169893275 | | |
| K01783 | rpe | ribulose-phosphate 3-epimerase | 0.933935456 | 0.277498625 | 0.630100087 | 0.856771896 | | |
| K00855 | PRK | phosphoribulokinase | 0.782437327 | 0.329670435 | 0.649169348 | 0.517506393 | | |
| K01595 | ppc | phosphoenolpyruvate carboxylase | 0.54132475 | 0.717438543 | 0.702310659 | 0.785413057 | | |
| K01610 | E4.1.1.49 | phosphoenolpyruvate carboxykinase (ATP) | 0.835221721 | 0.540261439 | 0.487329851 | 0.434050032 | | |
| K00024 | mdh | malate dehydrogenase | 0.386079244 | 0.718573781 | 0.624646577 | 0.997243188 | | |
| K01006 | ppdK | pyruvate, orthophosphate dikinase | 0.850583595 | 0.322613255 | 0.835347134 | 0.107783641 | | |
| **Oxidative Phosphorylation** | | | | | | |  |  |
| K00330 | nuoA | NADH-quinone oxidoreductase subunit A | 0.627402802 | 0.454845579 | 0.5616381 | 0.42107699 | | |
| K00331 | nuoB | NADH-quinone oxidoreductase subunit B | 0.615133842 | 0.394559362 | 0.558861083 | 0.819822213 | | |
| K00332 | nuoC | NADH-quinone oxidoreductase subunit C | 0.404908648 | 0.191857827 | 0.621585266 | 0.195988604 | | |
| K00333 | nuoD | NADH-quinone oxidoreductase subunit D | 0.950665615 | 0.022535019 | 0.105943849 | 0.12885789 | | |
| K00334 | nuoE | NADH-quinone oxidoreductase subunit E | 0.620283563 | 0.139344567 | 0.439344611 | 0.0637966 | | |
| K00335 | nuoF | NADH-quinone oxidoreductase subunit F | 0.983695184 | 0.056899833 | 0.102600189 | 0.286095697 | | |
| K00336 | nuoG | NADH-quinone oxidoreductase subunit G | 0.383888599 | 0.568584088 | 0.324599523 | 0.559552528 | | |
| K00337 | nuoH | NADH-quinone oxidoreductase subunit H | 0.755739954 | 0.291215022 | 0.048836034 | 0.291263406 | | |
| K00338 | nuoI | NADH-quinone oxidoreductase subunit I | 0.63575856 | 0.046365034 | 0.035935822 | 0.030132154 | | |
| K00339 | nuoJ | NADH-quinone oxidoreductase subunit J | 0.385106413 | 0.898257123 | 0.320196964 | 0.522329927 | | |
| K00340 | nuoK | NADH-quinone oxidoreductase subunit K | 0.411871048 | 0.983529807 | 0.475768283 | 0.229685919 | | |
| K00341 | nuoL | NADH-quinone oxidoreductase subunit L | 0.645473072 | 0.50972188 | 0.667683953 | 0.977338938 | | |
| K00342 | nuoM | NADH-quinone oxidoreductase subunit M | 0.429764017 | 0.916652621 | 0.334924931 | 0.923194079 | | |
| K00343 | nuoN | NADH-quinone oxidoreductase subunit N | 0.229335504 | 0.773885538 | 0.875000456 | 0.73926949 | | |
| K00356 | E1.6.99.3 | NADH dehydrogenase | 0.569374127 | 0.299494511 | 0.8426783 | 0.665797391 | | |
| K00239 | sdhA | succinate dehydrogenase, flavoprotein subunit | 0.869571418 | 0.455090663 | 0.705530223 | 0.891963068 | | |
| K00240 | sdhB | succinate dehydrogenase, iron-sulfur subunit | 0.573483978 | 0.225134228 | 0.607863755 | 0.541838967 | | |
| K00241 | sdhC | succinate dehydrogenase, cytochrome b subunit | 0.149223003 | 0.003703596 | 0.012390378 | 0.085897225 | | |
| K00242 | sdhD | succinate dehydrogenase, membrane anchor subunit | 0.665666151 | 0.07548327 | 0.524076274 | 0.292746538 | | |
| K00410 | fbcH | ubiquinol-cytochrome c reductase cytochrome b/c1 | 0.064429879 | 0.700536048 | 0.602969084 | 0.052268316 | | |
| K00411 | UQCRFS1 | ubiquinol-cytochrome c reductase iron-sulfur subunit | 0.583207483 | 0.565012132 | 0.800189472 | 0.223110356 | | |
| K00412 | CYTB | ubiquinol-cytochrome c reductase cytochrome b | 0.504862495 | 0.517332329 | 0.592889297 | 0.894986028 | | |
| K00413 | CYC1 | ubiquinol-cytochrome c reductase cytochrome c1 | 0.512083114 | 0.525667961 | 0.928041378 | 0.549088415 | | |
| K00425 | cydA | cytochrome d ubiquinol oxidase subunit I | 0.241236047 | 0.671489173 | 0.320997493 | 0.812843559 | | |
| K00426 | cydB | cytochrome d ubiquinol oxidase subunit II | 0.918270125 | 0.649973511 | 0.03377127 | 0.437968169 | | |
| K00404 | ccoN | cytochrome c oxidase cbb3-type subunit I | 0.020437306 | 0.00579224 | 0.037877206 | 0.322899123 | | |
| K00405 | ccoO | cytochrome c oxidase cbb3-type subunit II | 0.156046788 | 0.150197319 | 1 | 1 | | |
| K00406 | ccoP | cytochrome c oxidase cbb3-type subunit III | 3.35706E-15 | 1.46867E-13 | 3.62713E-11 | 0.719939892 | | |
| K02258 | COX11 | cytochrome c oxidase assembly protein subunit 11 | 0.230882959 | 0.101822645 | 0.409640287 | 0.03713762 | | |
| K02259 | COX15 | cytochrome c oxidase assembly protein subunit 15 | 0.655170882 | 0.952525083 | 0.574904284 | 0.140604474 | | |
| K02274 | coxA | cytochrome c oxidase subunit I | 0.222109353 | 0.192671394 | 0.403388657 | 0.251818655 | | |
| K02275 | coxB | cytochrome c oxidase subunit II | 0.143431048 | 0.002947011 | 0.024681009 | 0.838743238 | | |
| K02276 | coxC | cytochrome c oxidase subunit III | 0.083296375 | 0.000114843 | 0.000784781 | 0.832396768 | | |
| K02301 | cyoE | protoheme IX farnesyltransferase | 0.171855713 | 0.818441538 | 0.996890782 | 0.928279865 | | |
| **Pentose Phosphate** | | | | | | |  |  |
| K00033 | PGD | 6-phosphogluconate dehydrogenase | 0.467005578 | 0.921602969 | 0.686543784 | 0.636788048 | | |
| K00036 | G6PD | glucose-6-phosphate 1-dehydrogenase | 0.786535393 | 0.780208495 | 0.55060725 | 0.554513591 | | |
| K00615 | E2.2.1.1 | transketolase | 0.665734991 | 0.152506651 | 0.468529747 | 0.310901161 | | |
| K00616 | E2.2.1.2 | transaldolase | 0.251678473 | 0.883215508 | 0.899678365 | 0.867486405 | | |
| K00850 | pfkA | 6-phosphofructokinase 1 | 0.303353405 | 0.311573261 | 0.383002749 | 0.367563673 | | |
| K00948 | PRPS | ribose-phosphate pyrophosphokinase | 0.472136669 | 0.136780274 | 0.854003154 | 0.588721971 | | |
| K01053 | E3.1.1.17 | gluconolactonase | 0.064429879 | 0.751609199 | 0.464248268 | 0.322899123 | | |
| K01057 | PGLS | 6-phosphogluconolactonase | 0.07940542 | 0.583970575 | 0.777305913 | 0.514388829 | | |
| K01619 | deoC | deoxyribose-phosphate aldolase | 0.805093113 | 0.816793079 | 0.780512918 | 0.379431127 | | |
| K01623 | ALDO | fructose-bisphosphate aldolase, class I | 0.929544491 | 0.475210628 | 0.745875289 | 0.792346839 | | |
| K01624 | FBA | fructose-bisphosphate aldolase, class II | 0.886483257 | 0.096113297 | 0.362075073 | 0.421482444 | | |
| K16370 | pfkB | 6-phosphofructokinase 2 | 0.011070506 | 0.150197319 | 0.281628137 | 1 | | |
| K01783 | rpe | ribulose-phosphate 3-epimerase | 0.933935456 | 0.277498625 | 0.630100087 | 0.856771896 | | |
| K01807 | rpiA | ribose 5-phosphate isomerase A | 0.702621308 | 0.623144131 | 0.292351797 | 0.169893275 | | |
| K01810 | GPI | glucose-6-phosphate isomerase | 0.826738541 | 0.187219059 | 0.433727944 | 0.179159461 | | |
| K01835 | pgm | phosphoglucomutase | 0.70136452 | 0.13611734 | 0.250839638 | 0.297136627 | | |
| K11645 | fbaB | fructose-bisphosphate aldolase, class I | 0.196060593 | 0.142624261 | 0.702208081 | 0.125542348 | | |
| K15778 | pmm-pgm | phosphomannomutase / phosphoglucomutase | 0.608219225 | 0.62295052 | 0.836046361 | 0.488464314 | | |
| **Glycolysis/Gluconeogenesis** | | | | | | |  |  |
| K00121 | frmA, ADH5, adhC | S-(hydroxymethyl)glutathione dehydrogenase | 0.387412741 | 0.404547289 | 0.84106984 | 0.052268316 | | |
| K00128 | E1.2.1.3 | aldehyde dehydrogenase (NAD+) | 0.964655547 | 0.929963558 | 0.962442156 | 0.53283444 | | |
| K00134 | GAPDH, gapA | glyceraldehyde 3-phosphate dehydrogenase | 0.806695131 | 0.263306312 | 0.431495225 | 0.562658954 | | |
| K00161 | PDHA, pdhA | pyruvate dehydrogenase E1 component alpha subunit | 0.978428862 | 0.514792131 | 0.726606309 | 0.539253465 | | |
| K00162 | PDHB, pdhB | pyruvate dehydrogenase E1 component beta subunit | 0.314725216 | 0.253285611 | 0.585118167 | 0.279964047 | | |
| K00163 | aceE | pyruvate dehydrogenase E1 component | 0.365725487 | 0.392197449 | 0.453213887 | 0.324790689 | | |
| K00382 | DLD, 1pd, pdhD | dihydrolipoamide dehydrogenase | 0.765204189 | 0.863294099 | 0.950668202 | 0.338925658 | | |
| K00627 | DLAT, aceF, pdhC | pyruvate dehydrogenase E2 component | 0.392730033 | 0.497913003 | 0.410619398 | 0.052288729 | | |
| K00845 | glk | glucokinase | 0.1214267 | 0.422478329 | 0.293198165 | 0.126330689 | | |
| K00850 | pfkA, PFK | 6-phosphofructokinase 1 | 0.303353405 | 0.311573261 | 0.383002749 | 0.367563673 | | |
| K00873 | PK, pyk | pyruvate kinase | 0.59987912 | 0.502010233 | 0.558793955 | 0.287837104 | | |
| K00886 | ppgK | polyphosphate glucokinase | 1 | 0.150197319 | 1 | 0.180915616 | | |
| K00927 | PGK, pgk | phosphoglycerate kinase | 0.866728723 | 0.074938302 | 0.535857894 | 0.264735341 | | |
| K01596 | E4.1.1.32 | phosphoenolpyruvate carboxykinase (GTP) | 0.387412741 | 0.700536048 | 0.07963014 | 0.322899123 | | |
| K01610 | E4.1.1.49 | phosphoenolpyruvate carboxykinase (ATP) | 0.835221721 | 0.540261439 | 0.487329851 | 0.434050032 | | |
| K01623 | ALDO | fructose-bisphosphate aldolase, class I | 0.929544491 | 0.475210628 | 0.745875289 | 0.792346839 | | |
| K01624 | FBA | fructose-bisphosphate aldolase, class II | 0.886483257 | 0.096113297 | 0.362075073 | 0.421482444 | | |
| K01689 | ENO | enolase | 0.924899037 | 0.39903512 | 0.628119056 | 0.884863439 | | |
| K01792 | E5.1.3.15 | glucose-6-phosphate 1-epimerase | 0.708763457 | 0.434729169 | 0.932288716 | 0.664409351 | | |
| K01803 | TPI | triosephosphate isomerase (TIM) | 0.565863077 | 0.481389079 | 0.876011556 | 0.399579239 | | |
| K01810 | GPI | glucose-6-phosphate isomerase | 0.826738541 | 0.187219059 | 0.433727944 | 0.179159461 | | |
| K01834 | PGAM | 2,3-bisphosphoglycerate-dependent phosphoglycerate mutase | 0.571520453 | 0.318938709 | 0.805913291 | 0.816931338 | | |
| K01835 | pgm | phosphoglucomutase | 0.70136452 | 0.13611734 | 0.250839638 | 0.297136627 | | |
| K01895 | ACSS | acetyl-CoA synthetase | 0.674972207 | 0.638817204 | 0.986772216 | 0.847934837 | | |
| K11645 | fbaB | fructose-bisphosphate aldolase, class I | 0.196060593 | 0.142624261 | 0.702208081 | 0.125542348 | | |
| K13953 | adhP | alcohol dehydrogenase, propanol-preferring | 0.210222336 | 0.834375885 | 0.748577449 | 0.548097287 | | |
| K15633 | gpmI | 2,3-bisphosphoglycerate-independent phosphoglycerate mutase | 0.160739802 | 0.335895982 | 0.440922474 | 0.623548364 | | |
| K15634 | gpmB | probable phosphoglycerate mutase | 0.531693288 | 0.817731351 | 0.177627881 | 0.30514734 | | |
| K15635 | apgM | 2,3-bisphosphoglycerate-independent phosphoglycerate mutase | 0.740062368 | 0.087000704 | 0.119127939 | 0.683737801 | | |
| K15778 | pmm-pgm | phosphomannomutase / phosphoglucomutase | 0.608219225 | 0.62295052 | 0.836046361 | 0.488464314 | | |
| K16370 | pfkB | 6-phosphofructokinase 2 | 0.011070506 | 0.150197319 | 0.281628137 | 1 | | |
| **Tricarboxylic Acid Cycle (TCA)** | | | | | | | |  |
| K00024 | mdh | malate dehydrogenase | 0.386079244 | 0.718573781 | 0.624646577 | 0.997243188 | | |
| K00030 | IDH3 | isocitrate dehydrogenase (NAD+) | 0.048885637 | 1 | 0.281628137 | 0.104303132 | | |
| K00031 | IDH1, IDH2, icd | isocitrate dehydrogenase | 0.736091878 | 0.924522279 | 0.683873895 | 0.80330322 | | |
| K00161 | PDHA | pyruvate dehydrogenase E1 component alpha subunit | 0.978428862 | 0.514792131 | 0.726606309 | 0.539253465 | | |
| K00162 | PDHB | pyruvate dehydrogenase E1 component beta subunit | 0.314725216 | 0.253285611 | 0.585118167 | 0.279964047 | | |
| K00163 | aceE | pyruvate dehydrogenase E1 component | 0.365725487 | 0.392197449 | 0.453213887 | 0.324790689 | | |
| K00164 | OGDH, sucA | 2-oxoglutarate dehydrogenase E1 component | 0.560061174 | 0.865390066 | 0.976977523 | 0.930463436 | | |
| K00239 | sdhA, frdA | succinate dehydrogenase, flavoprotein subunit | 0.869571418 | 0.455090663 | 0.705530223 | 0.891963068 | | |
| K00240 | sdhB, frdB | succinate dehydrogenase, iron-sulfur subunit | 0.573483978 | 0.225134228 | 0.607863755 | 0.541838967 | | |
| K00241 | sdhC, frdC | succinate dehydrogenase, cytochrome b subunit | 0.149223003 | 0.003703596 | 0.012390378 | 0.085897225 | | |
| K00242 | sdhD, frdD | succinate dehydrogenase , membrane anchor subunit | 0.665666151 | 0.07548327 | 0.524076274 | 0.292746538 | | |
| K00382 | DLD, lpd, pdhD | dihydrolipoamide dehydrogenase | 0.765204189 | 0.863294099 | 0.950668202 | 0.338925658 | | |
| K00627 | DLAT, aceF, pdhC | pyruvate dehydrogenase E2 component | 0.392730033 | 0.497913003 | 0.410619398 | 0.052288729 | | |
| K00658 | DLST, sucB | 2-oxoglutarate dehydrogenase E2 component | 0.578852586 | 0.774909619 | 0.971174018 | 0.411620119 | | |
| K01596 | E4.1.1.32 pckA, PEPCK | phosphoenolpyruvate carboxykinase (GTP) | 0.387412741 | 0.700536048 | 0.07963014 | 0.322899123 | | |
| K01610 | E4.1.1.49 pckA | phosphoenolpyruvate carboxykinase (ATP) | 0.835221721 | 0.540261439 | 0.487329851 | 0.434050032 | | |
| K01647 | CS | citrate synthase | 0.304026173 | 0.504277569 | 0.943972114 | 0.554292118 | | |
| K01679 | E4.2.1.2B fumB | fumarate hydratase, class II | 0.319849006 | 0.197042716 | 0.445281242 | 0.518085938 | | |
| K01681 | ACO, acnA | aconitate hydratase | 0.337015074 | 0.560941625 | 0.519313442 | 0.455514617 | | |
| K01902 | sucD | succinyl-CoA synthetase alpha subunit | 0.393901029 | 0.889402425 | 0.918362007 | 0.70399687 | | |
| K01903 | sucC | succinyl-CoA synthetase beta subunit | 0.35088579 | 0.333913483 | 0.789282744 | 0.871599501 | | |

**Table SI-3**. Assigned organisms to the differentially expressed genes (p<0.05 and p<0.1) on selected pathways aligned to NCBI RefSeq database.

| **KO** | **Gene** | **Contig** | **Feature** | **Best BLAST hit in NCBI RefSeq^*^** |
| --- | --- | --- | --- | --- |
| K00376 | nosZ | c1446 | F_06367 | No hits found |
| K00368 | nirK | c1536 | F_06649 | No hits found |
|  |  | c4269 | F_12728 | *Nitrobacter winogradskyi*, gi\|75674199\|ref\|NC_007406.1\|:c2881015-2880044, 934/972 (97%), 0/972(0%) |
|  |  | rep_c8077 | F_16938 | *Nitrosospira multiformis*, gi\|82701135\|ref\|NC_007614.1\|:c3056580-3054868, 1464/1719 (86%) |
| K04561 | norB | c4877 | F_13713 | No hits found |
| K02305 | norC | c4098 | F_12417 | *Nitrobacter winogradskyi*, gi\|75674199\|ref\|NC_007406.1\|:1284973-1288830, 2091/2203 (95%), 9/2203 (0%) |
| K04748 | norQ | c736 | F_03740 | *Nitrobacter winogradskyi*, gi\|75674199\|ref\|NC_007406.1\|:c2164769-2162409, 1481/1548 (96%), 5/1548 (0%) |
| K00373 | narJ | c40 | F_00288 | *Nitrobacter winogradskyi*, gi\|75674199\|ref\|NC_007406.1\|:860039-863683,3445/3648 (95%), 6/3648 (0%) |
| K00374 | narI | c1881 | F_07698 | No hits found |
| K00261 | GLUD1_2 | c251 | F_01519 | *Nitrobacter winogradskyi*, gi\|75674199\|ref\|NC_007406.1\|:c2496340-2493245, 2950/3098 (96%), 12/3098 (0%) |
| K00266 | gltD | c14705 | F_28382 | No hits found |
|  |  | c2097 | F_08312 | *Nitrobacter winogradskyi*, gi\|75674199\|ref\|NC_007406.1\|:46476-48056, 1491/1559 (96%), 5/1559 (0%) |
|  |  | c2286 | F_08793 | No hits found |
| K01674 | cah | rep_c8181 | F_20306 | *Nitrobacter hamburgensis*, gi\|92115633\|ref\|NC_007964.1\|:4092467-4093966, 1318/1504 (88%), 26/1504 (1%) |
| K01725 | cynS | c1817 | F_07533 | *Nitrobacter winogradskyi*, gi\|75674199\|ref\|NC_007406.1\|:1436341-1436829, 468/489 (96%), 0/489 (0%) |
|  |  | c10750 | F_23551 | No hits found |
| K01673 | cynT, can | c1661 | F_07047 | *Nitrobacter hamburgensis*, gi\|92109250\|ref\|NC_007959.1\|:c172634-172266, 319/359 (89%), 6/359 (1%) |
|  |  | rep_c10708 | F_23493 | *Nitrobacter winogradskyi*, gi\|75674199\|ref\|NC_007406.1\|:2263467-2266580, 411/434 (95%), 2/434 (0%) |
|  |  | c2288 | F_08800 | *Nitrobacter winogradskyi*, gi\|75674199\|ref\|NC_007406.1\|:732820-734415, 961/1058 (91%), 15/1058 (1%) |
|  |  | c2516 | F_09334 | *Nitrobacter winogradskyi*, gi\|75674199\|ref\|NC_007406.1\|:92582-93802, 1139/1224 (94%), 6/1224 (0%) |
|  |  | c2754 | F_09816 | *Nitrobacter hamburgensis*, gi\|92115633\|ref\|NC_007964.1\|:3648075-3648815, 660/711 (93%), 1/711 (0%) |
|  |  | rep_c8107 | F_18546 | *Bordetella bronchiseptica*, gi\|412337338\|ref\|NC_019382.1\|:c2454798-2454172, 426/528 (81%), 20/528 (3%) |
|  |  | rep_c8123 | F_19258 | *Rubrivivax gelatinosus*, gi\|383755859\|ref\|NC_017075.1\|:c1138135-1136741, 235/283 (84%), 18/283 (6%) |
| K15876 | nrfH | c92 | F_00619 | No hits found |
| K01602 | cbbS | c1960 | F_07932 | No hits found |
|  |  | c3295 | F_10939 | *Nitrobacter winogradskyi*, gi\|75674199\|ref\|NC_007406.1\|:c3165853-3164384, 1174/1200 (98%), 0/1200 (0%) |
|  |  | rep_c8083 | F_17174 | *Nitrosospira multiformis*, gi\|82701135\|ref\|NC_007614.1\|:c792974-789801, 2489/3149 (80%), 111/3149 (3%) |
|  |  | rep_c8136 | F_19752 | *Nitrosomonas sp. Is79A3*, gi\|339481522\|ref\|NC_015731.1\|:c1464502-1462526, 1524/1858 (83%), 65/1858 (3%) |
| K01601 | cbbL | rep_c8083 | F_17175 | *Nitrosospira multiformis*, gi\|82701135\|ref\|NC_007614.1\|:c1132827-1130887, 1502/1870 (81%), 74/1870 (3%) |
|  |  | c1959 | F_07931 | *Nitrobacter winogradskyi*, gi\|75674199\|ref\|NC_007406.1\|:c2174681-2173260, 1370/1423 (97%), 2/1423 (0%) |
|  |  | rep_c8136 | F_19751 | no hits found |
| K00927 | pgk | c766 | F_03884 | no hits found |
|  |  | c1736 | F_07301 | *Nitrobacter winogradskyi*, gi\|75674199\|ref\|NC_007406.1\|:2970244-2971251, 971/1008 (97%), 0/1008 (0%) |
|  |  | rep_c8098 | F_18117 | *Nitrosospira multiformis*, gi\|82701135\|ref\|NC_007614.1\|:388273-390942, 2097/2578 (82%), 56/2578 (2%) |
| K01624 | FBA | rep_c8098 | F_18119 | *Nitrosospira multiformis*, gi\|82701135\|ref\|NC_007614.1\|:c429038-427023, 1637/2041 (81%), 50/2041 (2%) |
|  |  | c6950 | F_15823 | No hits found |
| K00333 | NuoD | c303 | F_01818 | *Nitrobacter winogradskyi*, gi\|75674199\|ref\|NC_007406.1\|:c2067024-2065699,1264/1331 (95%), 6/1331 (0%) |
|  |  | rep_c8077 | F_16924 | *Nitrosospira multiformis*, gi\|82701135\|ref\|NC_007614.1\|:1324193-1327771, 2845/3584 (80%), 101/3584 (2%) |
| K00335 | NuoF | c58 | F_00384 | no hits found |
|  |  | rep_c8077 | F_16922 | *Nitrosospira multiformis*, gi\|82701135\|ref\|NC_007614.1\|:1324193-1327771, 2845/3584 (80%), 101/3584 (2%) |
| K00337 | NuoH | c1422 | F_06274 | No hits found |
|  |  | rep_c8077 | F_16920 | *Nitrosospira multiformis*, gi\|82701135\|ref\|NC_007614.1\|:1324193-1327771, 2845/3584 (80%), 101/3584 (2%) |
| K00338 | NuoI | c90 | F_00584 | *Nitrobacter winogradskyi*, gi\|75674199\|ref\|NC_007406.1\|:c2055143-2053470, 1597/1674 (96%), 4/1674 (0%) |
|  |  | c5095 | F_14080 | No hits found |
|  |  | rep_c8077 | F_16918 | *Nitrosospira multiformis*, gi\|82701135\|ref\|NC_007614.1\|:1324193-1327771, 2845/3584 (80%), 101/3584 (2%) |
| K00356 | E1.6.99.3 | c734 | F_03729 | *Nitrobacter hamburgensis*, gi\|92115633\|ref\|NC_007964.1\|:c1326090-1325236, 215/236 (92%), 5/236 (2%) |
|  |  | c2043 | F_08165 | *Nitrobacter winogradskyi*, gi\|75674199\|ref\|NC_007406.1\|:192738-193544, 771/808 (96%), 2/808 (0%) |
|  |  | rep_c8152 | F_20047 | *Nitrosospira multiformis*, gi\|82701135\|ref\|NC_007614.1\|:2795836-2797179, 1074/1357 (80%), 46/1357 (3%) |
|  |  | c13156 | F_26818 | No hits found |
| K02275 | coxB | c958 | F_04614 | *Salinibacter ruber*, gi\|294505815\|ref\|NC_014032.1\|:c2673852-2671978, 597/815 (74%), 61/815 (7%), 1e-60 |
|  |  | rep_c8210 | F_20375 | *Nitrobacter winogradskyi*, gi\|75674199\|ref\|NC_007406.1\|:847441-849060, 1593/1623 (99%), 4/1623 (0%) |
|  |  | rep_c8085 | F_17484 | *Nitrosospira multiformis*, gi\|82701135\|ref\|NC_007614.1\|:540160-542055, 1578/1901 (84%), 20/1901 (1%) |
| K02258 | COX11 | rep_c8085 | F_17486 | *Nitrosospira multiformis*, gi\|82701135\|ref\|NC_007614.1\|:540160-542055, 1578/1901(84%), 20/1901 (1%) |
|  |  | c4478 | F_13087 | *Nitrobacter winogradskyi*, gi\|75674199\|ref\|NC_007406.1\|:850993-851847, 837/856 (98%), 2/856 (0%) |
|  |  | c5101 | F_14096 | *Nitrobacter winogradskyi*, gi\|75674199\|ref\|NC_007406.1\|:849000-850061, 996/1064 (94%), 13/1064 (1%) |
| K02276 | coxC | c4478 | F_13086 | *Nitrobacter winogradskyi*, gi\|75674199\|ref\|NC_007406.1\|:850993-851847, 837/856 (98%), 2/856 (0%) |
|  |  | rep_c8086 | F_17488 | *Nitrosospira multiformis*, gi\|82701135\|ref\|NC_007614.1\|:195918-197498, 1329/1587 (84%), 38/1587 (2%) |
|  |  | rep_c8168 | F_20215 | *Nitrosospira multiformis*, gi\|82701135\|ref\|NC_007614.1\|:c2030599-2029010, 1333/1562 (86%), 23/1562 (1%) |
| K00404 | ccoN | c104 | F_00686 | *Gemmatimonas aurantiaca*, gi\|226225406\|ref\|NC_012489.1\|:4432645-4434903, 909/1205 (76%), 81/1205 (6%) |
| K00241 | sdhC | c971 | F_04661 | *Nitrobacter winogradskyi*, gi\|75674199\|ref\|NC_007406.1\|:3036425-3038248, 1708/1827 (94%), 4/1827 (0%) |
|  |  | rep_c8128 | F_19487 | *Nitrosospira multiformis*, gi\|82701135\|ref\|NC_007614.1\|:c973077-971626, 1151/1481 (78%), 64/1481 (4%) |
| K00242 | sdhD | c2146 | F_08444 | *Nitrobacter winogradskyi*, gi\|75674199\|ref\|NC_007406.1\|:c2813699-2812821, 820/872 (95%), 0/872 (0%) |
|  |  | rep_c8128 | F_19486 | *Nitrosospira multiformis*, gi\|82701135\|ref\|NC_007614.1\|:c973077-971626, 1151/1481 (78%), 64/1481 (4%) |
| K00406 | ccoP | c1446 | F_06368 | No hits found |
| K00334 | nuoE | c303 | F_01820 | *Nitrobacter winogradskyi*, gi\|75674199\|ref\|NC_007406.1\|:c2065694-2063619, 1976/2080 (95%), 9/2080 (0%) |
|  |  | c684 | F_03518 | No hits found |
|  |  | rep_c8077 | F_16923 | *Nitrosospira multiformis*, gi\|82701135\|ref\|NC_007614.1\|:1324193-1327771, 2845/3584 (80%), 101/3584 (2%) |
| K00410 | fbcH | c728 | F_03697 | *Nitrobacter winogradskyi*, gi\|75674199\|ref\|NC_007406.1\|:c2843420-2841237, 2049/2150 (96%), 0/2150 (0%) |
| K00426 | cydB | c1628 | F_06960 | No hits found |
| K01053 | E.3.1.1.17 | c489 | F_02660 | No hits found |
|  |  | c4778 | F_13565 | *Nitrobacter winogradskyi*, gi\|75674199\|ref\|NC_007406.1\|:c1135670-1134819, 727/760 (96%), 0/760 (0%) |
| K01057 | PGLS | c2464 | F_09207 | *Nitrobacter winogradskyi*, gi\|75674199\|ref\|NC_007406.1\|:158519-159523, 820/901 (92%), 7/901 (0%) |
|  |  | c1288 | F_05801 | *Nitrobacter winogradskyi*, gi\|75674199\|ref\|NC_007406.1\|:2872645-2874159, 1448/1516 (96%), 2/1516 (0%) |
|  |  | rep_c8104 | F_18416 | *Burkholderia sp*., gi\|377819444\|ref\|NC_016589.1\|:c2208563-2207433, 593/784 (76%), 60/784 (7%), 4e-88 |
| K00627 | DLAT | c336 | F_01932 | *Nitrobacter hamburgensis*, gi\|92115633\|ref\|NC_007964.1\|:1953895-1955328, 1290/1444 (90%), 33/1444 (2%) |
|  |  | rep_c8098 | F_18144 | *Nitrosospira multiformis*, gi\|82701135\|ref\|NC_007614.1\|:388273-390942, 2097/2578 (82%), 56/2578 (2%) |
|  |  | c14952 | F_28609 | No hits found |
|  |  | c1205 | F_05520 | *Nitrobacter winogradskyi*, gi\|75674199\|ref\|NC_007406.1\|:602193-604397, 2077/2209 (95%), 7/2209 (0%) |
| K00030 | IDH3 | rep_c8805 | F_21271 | *Nitrobacter winogradskyi*, gi\|75674199\|ref\|NC_007406.1\|:c3371245-3370265, 767/981 (79%), 74/981 (7%), 1e-156 |
| K01596 | E4.1.1.32 | c3691 | F_11679 | *Nitrobacter hamburgensis*, gi\|92115633\|ref\|NC_007964.1\|:2280230-2282329, 601/695 (87%), 12/695 (1%) |
| K00121 | frmA | c1081 | F_05096 | No hits found |
| K15635 | apgM | c1295 | F_05858 | No hits found |
| K16370 | pfkB | c6353 | F_15251 | No hits found |

**^*^** Organism with highest scoring BLAST hit to NCBI RefSeq database. In order: organism, accession number | RefSeq |: alignment positions on NCBI RefSeq entry, identity, gap, e-value (for alignment below 80% identity).

**Figure SI-1**. Bioinformatics workflow of metagenomics and metatranscriptomics analyses.

Shotgun Sequencing

(Meta-genome)

RNA-Sequencing

(Meta-transcriptome)

Filtering

Quality Control

*de novo* Assembly

Annotation

(KEGG, BLAST)

Filtering

Quality Control

rRNA Read Removal

Alignment

Read Count

Normalization

Differential

Gene Expression

Community Composition

(BLAST)

**Figure SI-2.** Heatmap of gene expression on (A) Pentose Phosphate, (B) Tricarboxylic Acid Cycle and (C) Glycolysis/Gluconeogenesis.

**A**. Pentose Phosphate

**B**. Tricarboxylic Acid Cycle

**C**. Glycolysis/Gluconeogenesis

**References**

1. KEGG. (Kyoto Encyclopedia of Genes and Genomes). Accessed November 27, 2014, <http://www.genome.jp/kegg-bin/show_organism?menu_type=pathway_maps&org=neu>.

2. KAAS. (Kyoto Encyclopedia of Genes and Genomes Automated Annotation Service). Accessed April 17, 2018,

<http://www.genome.jp/tools/kaas>

3. Huson DH, Auch AF, Qi J, Schuster SC. MEGAN analysis of metagenomic data. Genome Res. 2007;17(3):377-86.

4. Letunic I, Bork P. Interactive Tree Of Life v2: online annotation and display of phylogenetic trees made easy. Nucleic Acids Research. 2011;39(Web Server issue):W475-W8.

5. Langmead B, Salzberg SL. Fast gapped-read alignment with Bowtie 2. Nat Meth. 2012;9(4):357-9.

6. Robinson MD, McCarthy DJ, Smyth GK. edgeR: a Bioconductor package for differential expression analysis of digital gene expression data. Bioinformatics (Oxford, England). 2010;26(1):139-40.

7. Warnes GR, Bolker B, Bonebakker L, Gentleman R, Liaw WHA, Lumley T, et al. gplots: Various R Programming Tools for Plotting Data. 2014.

8. Burgin AJ, Hamilton SK. Have we overemphasized the role of denitrification in aquatic ecosystems? A review of nitrate removal pathways. Frontiers in Ecology and the Environment. 2007;5(2):89-96.

9. Einsle O, Stach P, Messerschmidt A, Klimmek O, Simon J, Kroger A, et al. Crystallization and preliminary X-ray analysis of the membrane-bound cytochrome c nitrite reductase complex (NrfHA) from Wolinella succinogenes. Acta crystallographica Section D, Biological crystallography. 2002;58(Pt 2):341-2.

10. Rodrigues ML, Oliveira T, Matias PM, Martins IC, Valente FMA, Pereira IAC, et al. Crystallization and preliminary structure determination of the membrane-bound complex cytochrome c nitrite reductase from Desulfovibrio vulgaris Hildenborough. Acta Crystallographica Section F: Structural Biology and Crystallization Communications. 2006;62(Pt 6):565-8.

11. Kern M, Volz J, Simon J. The oxidative and nitrosative stress defence network of Wolinella succinogenes: cytochrome c nitrite reductase mediates the stress response to nitrite, nitric oxide, hydroxylamine and hydrogen peroxide. Environmental Microbiology. 2011;13(9):2478-94.

12. Klotz MG, Stein LY. Nitrifier genomics and evolution of the nitrogen cycle. FEMS Microbiol Lett. 2008;278(2):146-56.

13. Rajeev L, Chen A, Kazakov AE, Luning EG, Zane GM, Novichkov PS, et al. Regulation of Nitrite Stress Response in Desulfovibrio vulgaris Hildenborough, a Model Sulfate-Reducing Bacterium. Journal of bacteriology. 2015;197(21):3400-8.

14. Ward BB. Nitrification: an Introduction and Overview of the State of the Field. In: Ward BB, Arp DJ, Klotz MG, editors. Nitrification. Washington, DC: ASM Press; 2011. p. 445.

15. Anderson PM, Sung YC, Fuchs JA. The cyanase operon and cyanate metabolism. FEMS Microbiol Rev. 1990;7(3-4):247-52.

16. Harano Y, Suzuki I, Maeda S, Kaneko T, Tabata S, Omata T. Identification and nitrogen regulation of the cyanase gene from the cyanobacteria Synechocystis sp. strain PCC 6803 and Synechococcus sp. strain PCC 7942. Journal of bacteriology. 1997;179(18):5744-50.

17. Sung YC, Fuchs JA. Identification and characterization of a cyanate permease in Escherichia coli K-12. Journal of bacteriology. 1989;171(9):4674-8.

18. Anderson AJ, Dawes EA. Occurrence, metabolism, metabolic role, and industrial uses of bacterial polyhydroxyalkanoates. Microbiol Rev. 1990;54(4):450-72.

19. Guilloton M, Karst F. Isolation and characterization of Escherichia coli mutants lacking inducible cyanase. J Gen Microbiol. 1987;133(3):645-53.

20. Ma Y, Sundar S, Park H, Chandran K. The effect of inorganic carbon on microbial interactions in a biofilm nitritation-anammox process. Water Res. 2015;70:246-54.

21. Jiang D, Khunjar WO, Wett B, Murthy SN, Chandran K. Characterizing the metabolic trade-off in Nitrosomonas europaea in response to changes in inorganic carbon supply. Environ Sci Technol. 2015;49(4):2523-31.

22. Gurevich A, Saveliev V, Vyahhi N, Tesler G. QUAST: quality assessment tool for genome assemblies. Bioinformatics. 2013;29(8):1072-5.
